# Polytherapy with a combination of three repurposed drugs (PXT3003) down-regulates *Pmp22* over-expression and improves myelination, axonal and functional parameters in models of CMT1A neuropathy

**DOI:** 10.1186/s13023-014-0201-x

**Published:** 2014-12-10

**Authors:** Ilya Chumakov, Aude Milet, Nathalie Cholet, Gwenaël Primas, Aurélie Boucard, Yannick Pereira, Esther Graudens, Jonas Mandel, Julien Laffaire, Julie Foucquier, Fabrice Glibert, Viviane Bertrand, Klaus-Armin Nave, Michael W Sereda, Emmanuel Vial, Mickaël Guedj, Rodolphe Hajj, Serguei Nabirotchkin, Daniel Cohen

**Affiliations:** Pharnext, 11, rue des Peupliers, 92130 Issy-Les-Moulineaux, France; Department of Neurogenetics, Max Planck Institute of Experimental Medicine, Hermann-Rein-Strasse 3, 37075 Göttingen, Germany; Department of Neurophysiology, University Medical Center, Robert Koch Str. 40, 37075 Göttingen, Germany

**Keywords:** Systems Biology, Repurposing, Combination therapy, Baclofen, Naltrexone, Sorbitol, Synergy, CMT1A, Low dose

## Abstract

Charcot-Marie-Tooth disease type 1A (CMT1A) is the most common inherited sensory and motor peripheral neuropathy. It is caused by *PMP22* overexpression which leads to defects of peripheral myelination, loss of long axons, and progressive impairment then disability. There is no treatment available despite observations that monotherapeutic interventions slow progression in rodent models. We thus hypothesized that a polytherapeutic approach using several drugs, previously approved for other diseases, could be beneficial by simultaneously targeting *PMP22* and pathways important for myelination and axonal integrity. A combination of drugs for CMT1A polytherapy was chosen from a group of authorised drugs for unrelated diseases using a systems biology approach, followed by pharmacological safety considerations. Testing and proof of synergism of these drugs were performed in a co-culture model of DRG neurons and Schwann cells derived from a *Pmp22* transgenic rat model of CMT1A. Their ability to lower *Pmp22* mRNA in Schwann cells relative to house-keeping genes or to a second myelin transcript (*Mpz*) was assessed in a clonal cell line expressing these genes. Finally *in vivo* efficacy of the combination was tested in two models: CMT1A transgenic rats, and mice that recover from a nerve crush injury, a model to assess neuroprotection and regeneration. Combination of (RS)-baclofen, naltrexone hydrochloride and D-sorbitol, termed PXT3003, improved myelination in the *Pmp22* transgenic co-culture cellular model, and moderately down-regulated *Pmp22* mRNA expression in Schwannoma cells. In both *in vitro* systems, the combination of drugs was revealed to possess synergistic effects, which provided the rationale for *in vivo* clinical testing of rodent models. In *Pmp22* transgenic CMT1A rats, PXT3003 down-regulated the *Pmp22 to Mpz* mRNA ratio, improved myelination of small fibres, increased nerve conduction and ameliorated the clinical phenotype. PXT3003 also improved axonal regeneration and remyelination in the murine nerve crush model. Based on these observations in preclinical models, a clinical trial of PTX3003 in CMT1A, a neglected orphan disease, is warranted. If the efficacy of PTX3003 is confirmed, rational polytherapy based on novel combinations of existing non-toxic drugs with pleiotropic effects may represent a promising approach for rapid drug development.

**Electronic supplementary material:**

The online version of this article (doi:10.1186/s13023-014-0201-x) contains supplementary material, which is available to authorized users.

## Background

Charcot-Marie-Tooth disease (ORPHA166), although rare, is the most common hereditary peripheral neuropathy with an estimated prevalence of 1 in 2,500 [1]. Seventy disease genes have now been identified to cause defects primarily in long axons or myelinating Schwann cells [2]. Approximately half of the patients belong to the CMT1A subtype which is caused in the majority of cases by the duplication of a 1.5 megabase region of human chromosome 17 containing the myelin gene *PMP22* encoding the peripheral myelin protein of 22 kDa. Overexpression of this gene by 50% results in abnormal Schwann cell differentiation and dysmyelination [3], eventually leading to axonal loss and muscle wasting. This striking dosage sensitivity points to the important regulatory function of PMP22 protein both for integrity as well as the function of peripheral nerves. Tight reciprocal interactions of neurons and Schwann cells are essential for peripheral nervous system (PNS). Axons provide signalling clues necessary for normal differentiation of Schwann cells, while Schwann cells not only insulate growing axons, but also deliver trophic factors supporting neuronal functions [4]. We hypothesized that polytherapeutic intervention to treat CMT1A should include agents able both to normalise *PMP22* gene expression, and to improve axonal dysfunction. As a first step we identified possible targets for intervention, looking at signalling pathways. Then we selected compounds known as to interfere with these pathways, to finally test these compounds in a rat model of the disease.

## Methods

### Systems biology analysis

Genes associated with development of Charcot-Marie-Tooth disease were retrieved from OMIM database (Online Mendelian Inheritance in Man database: http://www.ncbi.nlm.nih.gov/omim).

The initial identification and analysis of the potentially affected CMT1A-relevant signalling pathways, integrating these genes, was made with the help of Ingenuity (https://analysis.ingenuity.com) and GeneGO (https://portal.genego.com) databases/bioinformatics tools and proprietary bioinformatics programs, with subsequent filtering and manual curation of the data. This initial analysis was complemented by manual updating of the most recent literature findings. The choice of the drugs was made through matching the emerging relevant targets using with the help of DrugBank, Reaxys and Integrity databases (http://www.drugbank.ca, http://www.elsevier.com/online-tools/reaxys, https://integrity.thomson-pharma.com). Final selection of candidate drugs for functional validation studies included also analysis of their pharmacological efficacy and safety profiles (https://www.medicinescomplete.com).

### Drugs

(RS)-baclofen (B5399), naltrexone hydrochloride (N3136) and D-sorbitol (S3889) were all obtained from Sigma-Aldrich. PXT3003 consisted in the combination of these 3 drugs.

### Co-cultures of sensory neurons and Schwann cells

15 days gestation pregnant Wild Type (WT) Sprague Dawley female rats, bred with CMT1A heterozygous rats [5], were killed by cervical dislocation and embryos (E15) were removed from the uterus. Rat Dorsal Root Ganglia (DRG) cultures were obtained as previously described [6-8] and performed at Neuronexperts laboratories (Marseille, France). The cultures were maintained in standard Neurobasal medium for 7 days to allow Schwann cells to populate sensory neurites. On day 7, the cultures were incubated with standard neuronal medium containing 50 μg/mL ascorbic acid (in order to initiate basal lamina formation and myelination) and drugs until 19 days. Our analyses of myelination were performed after 10–11 days of incubation. Three separate and independent cultures of DRG (from Transgenic (TG) embryos male rats) were performed with 6 replicates per condition.

### Immuno-staining

Cells were fixed by a cold solution of ethanol (95%) and acetic acid (5%) for 10 min, permeabilised, and blocked with PBS containing 0.1% saponin (Sigma) and 10% FBS (Invitrogen) for 15 min. Then, cells were incubated with a rabbit polyclonal antibody specific to Myelin Basic Protein (MBP) (Sigma-Aldrich; M3821). MBP staining was revealed with Alexa Fluor 568 goat anti-rabbit IgG (Molecular Probes). Nuclei were counter-stained with Hoechst (Sigma-Aldrich). 20 pictures per well were taken in the same conditions using the InCell Analyzer™ 1000 (GE Healthcare) with 20 × magnification. Analysis of total length of myelinated axonal segments and total number of nuclei was automatically performed with the InCell Analyzer Software. Total nuclei count showed consistency in all wells and at each time point of the experiment.

### RT4 schwannoma cultures

RT4-D6P2T cell line was provided by ATCC (CRL-2768, batch 3993689, mycoplasma-free). Cells were thawed and cultured at Neurofit facilities (Illkirch, France) in DMEM medium (ATCC) containing 10% Foetal Bovine Serum (FBS; ATCC) and 1% antibiotic-antimicotic mixture (Gibco), and maintained in a humidified incubator at 37°C in 5% CO_2_-95% air atmosphere. Two days later, cells were trypsinised and transferred to 12-well plates (Nunc) at 30,000 cells per well. After 48 h, culture medium was replaced by DMEM containing drugs without FBS. After 8 h of treatment, cells were washed and harvested in 100 μL Phosphate Buffer Saline (PBS), then centrifuged 10 min at 13,000 rpm (4°C). The supernatant was discarded and the pellet was stored at −80°C until use. Three experiments with two independent cultures of RT4 were performed and each condition was done in triplicate.

### Animals and housing

CMT1A transgenic rats initial colony was established by a couple of heterozygous transgenic rats belonging to the original source [5]. We used only male animals to reduce potential variability in our experiments [5]. Nevertheless, male rats are clinically indistinguishable from female CMT1A rats [9]. Animals were housed and maintained at Key-Obs (Orléans, France). Two to three animals were housed per type III polycarbonate cages (Techniplast, Italy) under standard conditions at constant temperature (22 ± 1.5°C), hygrometry (50 ± 25%), and lighting conditions (50 Lux in housing room and 13 Lux in experimental room) with a 12/12 h daylight cycle. CD-1 mice were provided by Janvier Labs (Saint-Berthevin, France). Mice were housed at the animal facility of Neurofit (Illkirch, France). They were group-housed (5–10 mice per cage) and maintained in a room with controlled temperature (21–22°C) and a reversed light–dark cycle (12h/12h). Animals had free access to food and tap water. Animal procedures were conducted in strict adherence to the EU Directive of September 22, 2010 (2010/63/UE). Only male animals were included in the experiments.

### Genotyping

#### Rat (E15) embryos

A piece of each E15 embryo head (3 mm^3^) was used for genotyping. DNA was extracted with the SYBR Green Extract-N-Amp tissue PCR kit (Sigma), then, quantitative PCR (qPCR) was performed using the 7500 fast RT-PCR system and analysed following the manufacturer’s instructions (Applied Biosystems). The gender of each embryo was determined using the male *Sry* [NM_012772] primers [10] (F: 5′-GAGAGAGGCACAAGTTGGC-3′; R: 5′-GCCTCCTGGAAAAAGGGCC-3′). The genotyping of each embryo was determined using the *Pmp22* [NM_008885] primers [5]. PCR was performed (20 s at 95°C, 45 cycles of 10 s at 95°C, 10 s at 65°C then 30 s at 72°C) with the 7500 fast RT-PCR system (Applied Biosystems).

#### CMT1A rats (aged 3 weeks)

Total DNA was isolated from ear biopsies by the single-step purification method with DNeasy blood & Tissue kit (Qiagen Gmbh) according to the manufacturer’s protocol. PCR was performed (10 min at 95°C, 45 cycles of 10 s at 95°C, 20 s at 64°C then 20 s at 72°C) with a rapid thermal cycler system (LightCycler® 480 II, 96 wells, Roche) using LightCycler® 480 SYBR Green I Master (Roche). Rat *Pmp22* gene DNA [NM_17037.1] was used as a quality control of DNA extraction (F: 5′-GACAAACCCCAGATGGCC-3′, R: 5′-CCGCAGCCACCAGCTATTGGT-3′). Mouse-specific *Pmp22* gene [NM_008885.2] PCR primers (F: 5′-GACAAACCCCAGACAGTTGA-3′; R: 5′-CAGGAGCCACCAGCTATTACT-3′) was used to identify transgenic rats. Primers were synthetised by Eurofins (MWG Operon, Germany).

### Sciatic nerve crush

Experiments were performed at Neurofit facilities. Sample sizes used for analyses were determined based on previous experience on assay variability. 4–5 week-old CD-1 male mice were anaesthetised using isoflurane (2.5–3% in air). The right thigh was shaved and the sciatic nerve was exposed at mid-thigh level (5 mm proximal to the bifurcation of the sciatic nerve) and crushed for 10 s twice with a microforceps (Holtex) with a 90° rotation between each crush. For sham operated animals, sciatic nerves were exposed but not crushed. Finally, the skin incision was secured with wound clips. Forty two days after the crush, the tibial nerve was taken out from 6 mice per group to perform morphometric analyses.

### Drug treatment

#### CMT1A rats

Stratification of the animals was based on the weight, the parent origin and the muscular performances obtained in behavioural tests (bar test and inclined plane test) performed one week before drug treatment. Randomization of animals was done to avoid bias in animal studies [11,12]. PXT3003 contained 30 μg/kg (RS)-baclofen, 3.5 μg/kg naltrexone hydrochloride and 1.05 mg/kg D-sorbitol. Drugs were dissolved in distilled water and were freshly prepared daily before each *per os* administration. PXT3003 was administered once daily by gavage with inox steel canula (Dominique Dutscher, 075486) in a volume of 1 mL/kg. At the dates of behavioural testing, gavages were performed after the test. Ages and treatment durations of rats are stated in the behaviour section.

#### CD-1 mice

Drugs were dissolved in distilled water and were freshly prepared daily before each *per os* administration. First administration of treatment (PXT3003: mix of (RS)-baclofen (60 or 600 μg/kg), naltrexone (7 or 70 μg/kg) and D-sorbitol (2.1 or 21 mg/kg)) was performed 30 min after the crush. From day 1 to day 42, administration was performed once daily. On test days, mice were treated 1.5 h before Compound Muscle Action Potential (CMAP) recording.

### *Pmp22* mRNA expression in RT4 cells and in sciatic nerves of CMT1A rats (9 weeks of treatment, 17 weeks of age)

Total RNA was isolated from RT4 cells using the RNeasy Micro Kit (Qiagen Gmbh) as described by the manufacturer’s protocol (Qiagen-RNeasy Micro Handbook), while total RNA was isolated from sciatic nerves using Qiazol (Qiagen Gmbh) followed by the single-step purification method with RNeasy Mini Kit (Qiagen Gmbh) as described by the manufacturer’s protocol (Qiagen-RNeasy Fibrous tissue Handbook). DNA contamination was removed by RNase-free DNase I (Qiagen-RNase-free DNase set 1,500 Kunits). RNA concentrations were estimated by NanoDrop ND-1000. RNA quality control was performed by Agilent RNA 6000 nano chips on Agilent 2100 Bioanalyzer. 80 ng of total RNA was reverse transcribed using SuperScript™ II Reverse Transcriptase with Oligo(dT)12–18 (Invitrogen), then, qPCR was performed with a rapid thermal cycler system (LightCycler® 480 II, Roche). Amplifications were performed (10 min at 95°C, 45 cycles of 10 s at 95°C, 40 s at 60°C then 10 s at 72°C) using LightCycler® 480 SYBR Green I Master (Roche). The sequences of the primers (synthesised by Eurofins) used for the Reverse Transcription quantitative PCR (RTqPCR) analyses were: *Rps9* [NM_031108.2]: F: 5′-ATCCGCCAACGTCACATC-3′ and R: 5′-CCGCCACCATAAGGAGAAC-3′, *Pmp22* rat and mouse [NM_17037.1] and [NM_008885.2]: F: 5′-TGTACCACATCCGCCTTGG-3′ and R: 5′-GAGCTGGCAGAAGAACAGGAAC-3′, *Mpz* [NM_017027.1]: F: 5′-TGTTGCTGCTGTTGCTCTTC-3′ and R: 5′-TTGTGAAATTTCCCCTTCTCC-3′, *Actb* [NM_031144.2]: F: 5′-CACCATGTACCCAGGCATT-3′ and R: 5′-ACTTGCGCTCAGGAGGAG-3′.

### Behaviour

Animals were tested in a random and blind manner for treatment and outcome measurements. Behavioural experiments and readouts (bar, inclined plane, hot plate, weight bearing and electrophysiological tests) were performed and validated at Key-Obs and Neurofit facilities by the examiners who were blinded for the treatment. Sample sizes used for behavioural analyses were also determined based on previous experience on assay variability [9,13].

#### CMT1A rats

Bar and inclined plane tests were performed on CMT1A rats after 9 weeks of treatment, 13–17 weeks of age.

*Bar test:* This test evaluated the muscular strength of the four paws and the equilibrium performances on a fixed rod. The rat was placed on its four paws on the middle of the wooden rod (diameter: 2.5 cm; length: 50 cm). The time spent on the bar (fall latency) in each trial and the number of falls were recorded. Five successive trials were performed (180 s max) [5].

*Inclined plane test:* The sliding apparatus [14] of 30 × 50 cm Plexiglas plane that could be inclined from an angle of 0° (horizontal) up to 60° was used. For each angle, two trials separated by 1 min were performed. Each rat was initially placed on the 25° inclined plane in the up-headed position (head-up orientation). From 30 min up to 1 h later, the same experiment was performed on a 35° inclined plane. Between the tests, rats were returned to their cages. The Plexiglas plane was cleaned after each trial. The performances of rats were evaluated by a 3-level score from 0 (no slide) to 3 (the rat slides to the bottom of the plane). The analysis was performed on the mean score at 25° and 35°.

*Hot plate:* The animal (after 4 months of treatment, 5 months of age) was placed into a glass cylinder on a hot plate adjusted to 48°C. The latency of the first reaction of the hind paw either left or right was recorded (paw lifting or licking, leaps or a jump to escape the heat). The cut-off time was set to 30 s [15].

Adverse events were monitored along animal experiments by assessment of the following parameters: loss of weight, lack of appetite, diarrhoea, sneezing, sniffling, laboured breathing, rough hair coat, hair loss, inactivity, abnormal grooming behaviour, aggressiveness.

#### CD-1 mice, hind limb weight bearing

Animals’ weight distribution on the four limbs was assessed on day 13 using the dynamic weight bearing test (BioSeb, France) [16]. This disability test consists in a continuous measurement of all pressure points applied by a freely moving animal, allowing a quantitative evaluation of the weight imbalance caused by pain. Each mouse was placed in an 11 × 11 × 22 cm cage with a 44 × 44 sensor cells grid on the floor. The pressure applied on sensor cells by animal’s paws was recorded at a 10 Hz frequency over a 5 min period. Pressure and surface detection thresholds were determined automatically for each animal by the Dynamic weight bearing 1.3.2 h software (Bioseb, France). After the manual attribution of each pressure point to the corresponding paw, the mean weight applied on each paw was calculated for surface ratio. Unilateral gait dysfunction was finally evaluated through the ipsi/contralateral hind paw weight and surface ratio.

### Electrophysiology

Electrophysiological studies and readouts were done and validated at Neurofit laboratories. Rats or mice were anaesthetised by 2.5–3% isoflurane-air mixture. CMAP was recorded by needle electrodes placed into the intrinsic foot muscles of the plantar surface (Keypoint electromyography, Medtronic, France). Subcutaneous monopolar needle electrodes (Medtronic, 9013R0312) were used for both stimulation and recording. 12.8 mA square wave pulses of 0.2 ms duration were used to stimulate sciatic nerve. For the CMT1A rats (after 8 months of treatment, 9 months of age), the latencies of CMAPs elicited by stimulation at both proximal (hip) and distal (hock) sites were measured. Motor Nerve Conduction Velocity (MNCV) was estimated by the time interval between two stimulation sites latencies relative to distance between these sites (with leg fully extended) [5,17]. For the CD-1 crushed mice (42 days of treatment), the right sciatic nerve (ipsilateral) was stimulated with single pulse applied at the sciatic notch. CMAP was recorded by needle electrodes placed at the gastrocnemius muscle. The amplitude of the action potential was determined 30 days after the crush.

### Nerve histology

Mice (42 days of treatment) or CMT1A rats (after 4 months of treatment, 5 months of age) were anaesthetised with intraperitoneal mixture of ketamine and xylazine (80 mg/kg, and 15 mg/kg, i.p. respectively). 5 mm segment of the right sciatic nerve of rats (just above the bifurcation of sciatic nerve into two branches, in contrast to other studies collecting sciatic nerves at their distal (1/3) ends [3]), or tibial nerve of mice were excised. Tissues were fixed in 4% glutaraldehyde in PBS (Sigma-Aldrich) at 4°C overnight. Then, nerve samples were rinsed with PBS at room temperature and post-fixed in 1% osmium tetroxide (EMS, France) for 1 h, rinsed in PBS and dehydrated in serial alcohol solutions (VWR Prolabo), and embedded in Epon. Embedded tissues were kept at 70°C during the 3 days of polymerisation. The tissue block was cut with a microtome (Microm, HM360). Transverse sections 5 μm thick were performed. Then, 1.5 μm cross section was performed and stained with 1% of toluidine blue (EMS) for 2 min, rinsed in PBS, dehydrated in serial alcohol solutions (95% and 100%) followed by xylene solution substitute (EMS) for 10 min at room temperature, and mounted in Eukitt (EMS).

### Morphometric analyses

Composite image of the entire nerve section of each sample was obtained using an optical microscope (Nikon optiphot-2) equipped with a digital camera (Nikon DS-Fi1). Morphometric analyses were performed with digital images using Image-Pro Plus software (Media Cybernetics Inc., USA), which computes the axonal and myelin pixel sizes after individualisation of each myelinated fibre via the grey level of toluidine blue-stained myelin sheath. These two parameters were used to calculate the number of myelinated axons and the g-ratio (ratio of the inner axonal diameter to the total outer).

### Statistical analyses

Statistical tests were two-tailed and conducted at a 5% significance level. Sample sizes used for behavioural analyses were defined on the basis of previous experience on assay variability [9,13]. Data distribution and within-group variation were preliminary assessed in order to guide our methodological choices. Statistical analyses were performed with Prism (http://www.graphpad.com/scientific-software/prism) and *R* (http://cran.r-project.org). We applied an Analysis of Variance (ANOVA) with Dunnett’s test for comparison of more than one experimental group to a reference and a Welch’s *t*-test for comparison of two experimental groups. These tests are described to be robust enough to overcome changes in distribution and variation [18,19]. We performed a combined analysis of all cultures and replicates of *in vitro* experimental data to assess myelination in DRG co-cultures after 10–11 days of treatment, and to study gene expression in RT4 schwannoma cells. In each *in vitro* experimental system (DRG and RT4), data were normalised to the non-treated vehicle control. In DRG co-cultures, synergy was assessed by drug combination analysis with isobologram and calculation of Combination Index (CI) that compares dosages for a given combination to those expected to obtain the same combination effect under a simple additive assumption, and offers a quantitative definition for additive effect (CI = 1), synergism (CI < 1), or antagonism (CI > 1) [20-23]. To study the superiority of PXT3003 to its single drugs in RT4 cells, an ANOVA model was fitted and the difference between the effect of PXT3003 and the effect of the most efficient drug (i.e. the drug having the largest effect when compared to control) was tested using a post-hoc test of contrast. Kaplan-Meier curves were analysed with a Cox model. Analyses were adjusted to the age of starting treatment when appropriate (bar test and inclined plane test). Statistical analysis of linear regression of g-ratio with respect to the axon diameter was performed on slope.

## Results

### Systems biology analysis and drug choice

**Figure 1:**
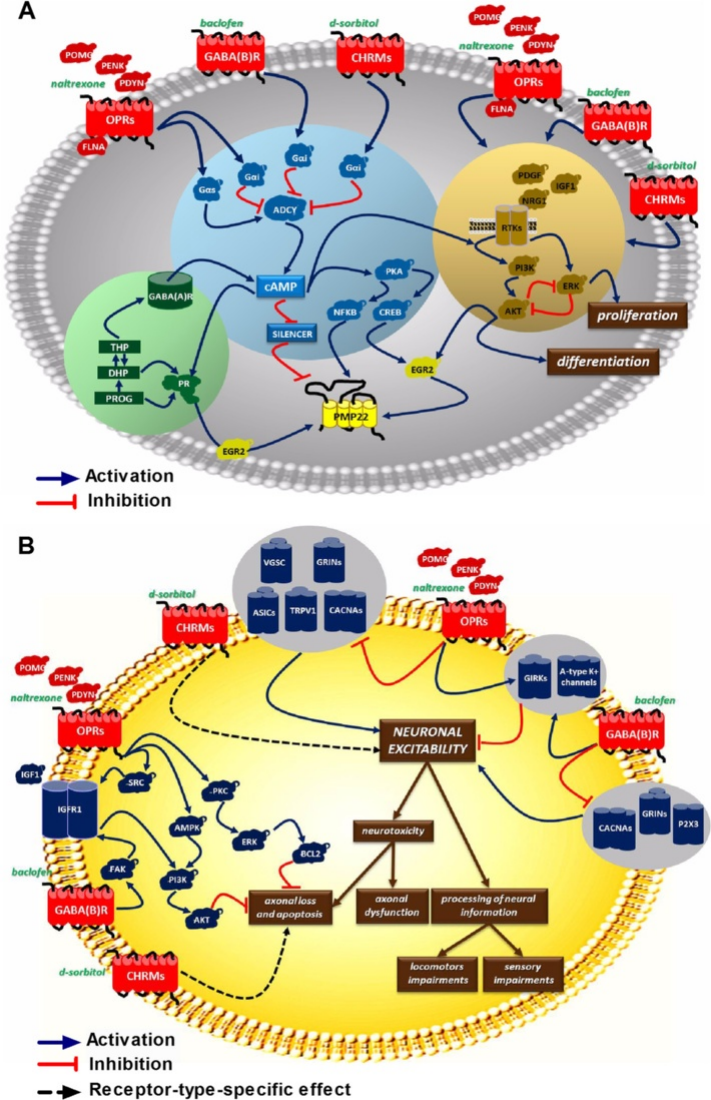
**Pharmacology network-based drug repurposing for CMT1A disease.** For detailed explanations and abbreviations, see Additional file 1. **(A)** Three principal pathways regulating expression of *PMP22* gene through extracellular GPCR signalling in Schwann cells. The functional interaction between cAMP pathway, neurosteroid-mediated signalling and PI3K-AKT/ERK kinase cascades, activated by receptor tyrosine kinases, control the expression of *PMP22* gene in Schwann cells. Individual drugs are shown in green. Blue shading: cAMP pathway; green shading: neurosteroid-mediated signalling; yellow shading: RTK/PI3K-AKT/ERK kinase cascades. **(B)** Cytoprotective and neuromodulator actions of PXT3003 drug combination in peripheral neurons. Dysfunction of CMT1A Schwann cells could affect membrane excitability of neuronal cells, leading to abnormal processing of neuronal information, cytotoxicity and axonal loss. GPCRs, modulated by PXT3003 drugs, are well-known regulators of neuronal excitability and pain sensation, are able to activate cytoprotective signalling pathways in different experimental settings, and could preserve functional integrity of peripheral neurons in CMT1 patients. Baclofen, naltrexone and sorbitol are shown in green. Red symbols: established or putative functional targets for PTX3003 drugs. Blue arrows: activation links; red lines: inhibition links; dashed lines: functional effect link is receptor-type specific.

As a first step of our discovery we performed a systematic analysis of available data to define a group of signalling pathways important for peripheral nerve structure and function affected in CMT1A disease (Figure 1 and Additional file 1). Among them, modular pathways known to affect myelin gene expression such as cAMP-dependent mechanisms, neurosteroid signalling and the Akt/Erk pathway were of particular interest [24-28] (Figure 1A and Additional file 1A). We hypothesised that these modules are integrated as a unified system that is influenced by G protein coupled receptors (GPCRs) leading to the differential regulation of genes for peripheral myelin proteins. Since PMP22 is not only a structural component of myelin, but may have signalling functions in Schwann cells, its transcriptional control could be different from “classical” myelin genes such as *MPZ*. The topology of these putative regulatory networks permitted us to suggest that drugs acting on different GPCRs could cause a more potent and robust influence when combined. Thus, we have focused on the drugs able to modify relevant branches of GPCR signalling. This class of compounds is functionally pleiotropic, acting on multiple pathways and is well represented in approved pharmacopeia. This fact permitted us to apply additional safety criteria for their selection. We also preferred drugs that could be important for other aspects of peripheral nerve physiology (Figure 1B and Additional file 1B) that are affected in CMT1A, particularly drugs potentially promoting neuronal protection. Eventually, three drugs – (RS)-baclofen, naltrexone and D-sorbitol – were chosen for testing in the relevant cellular and animal models of CMT1A.

The first drug, baclofen (BCL), is a specific agonist of GABA_B_ receptors. These receptors decrease the activity of adenylate cyclases and therefore reduce the levels of intracellular cAMP that positively regulate PMP22 expression [24,25,29-31]. GABA_B_ receptors are also abundant in neurons [32]. BCL is a safe drug currently used to treat spasticity. The second selected drug is the opioid receptor antagonist naltrexone (NTX). It is approved for treating drug addiction, and was shown at low non-toxic doses, to potentiate, rather than to block, cell signalling through opioid receptors that are coupled to inhibitory G alpha protein subunit (Gαi), thereby reducing intracellular levels of cAMP. NTX could mediate this action either through interaction with accessory protein Filamin A (FLNA) [33] or by induction of endogenous opioid agonists [34]. The third compound D-sorbitol (SRB), a natural metabolite playing an important role in the energy production/storage (polyol pathway), was chosen as another safe drug involved in processes that are deregulated in CMT1A. No specific receptor for SRB is currently known although muscarinic acetylcholine GPCRs, present both on neurons and Schwann cells and affecting cAMP levels, were reported to bind SRB with unexpected high affinity [35]. Moreover, SRB acting as chaperone might also improve PMP22 protein folding [36,37] that is impaired when overexpressed in CMT1A Schwann cells [38,39]. Importantly, we hypothesised that a combination of these drugs (PXT3003), by acting on different receptor systems, could decrease the toxic effects of the overexpression of *PMP22* gene and also improve downstream consequences on myelination and nerve function. This hypothesis was then tested in a battery of *ex vivo* and *in vivo* tests modelling different aspects of CMT1A disease.

### PXT3003 improves myelination *ex vivo*

**Figure 2:**
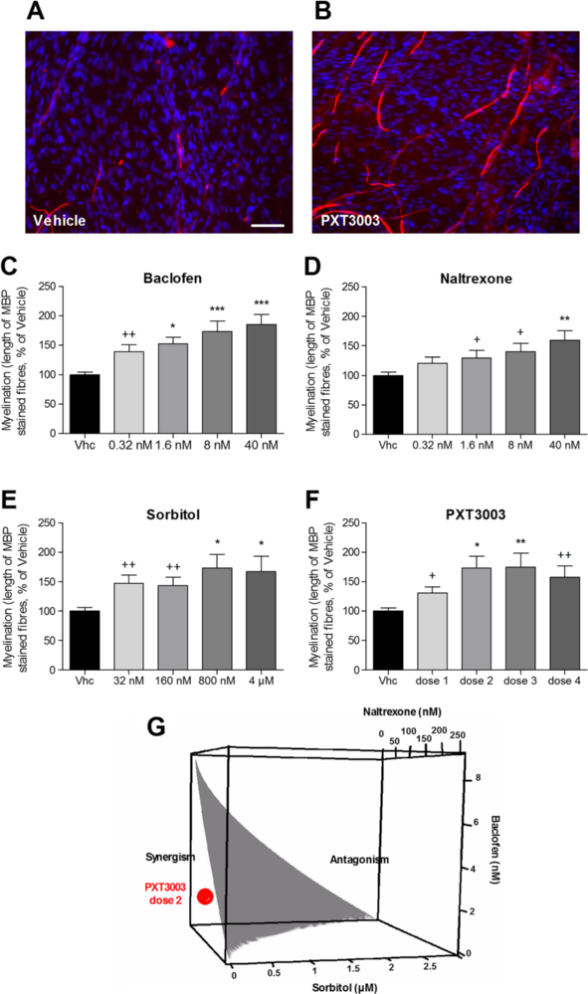
**BCL, NTX, SRB and their mix (PXT3003) improve myelination in DRG co-cultures. (A)** and **(B)** PXT3003 treatment (Dose 2) of myelinating DRG co-cultures of neurons and Schwann cells derived from CMT1A transgenic rats (TG) demonstrated an increased length of MBP-positive fibres (red staining in **(B)**) compared to the untreated control **(A)**. Scale bar: 56 μm. Blue staining: DAPI. **(C)** to **(F)** After 10–11 days of treatment of TG DRG co-cultures with single compounds BCL **(C)**, NTX **(D)**, SRB **(E)** or their PXT3003 **(F)** combination at four doses (Dose 1: 0.32 nM of BCL, 0.32 nM of NTX and 32 nM of SRB; Dose 2: 1.6 nM of BCL, 1.6 nM of NTX and 160 nM of SRB; Dose 3: 8 nM of BCL, 8 nM of NTX and 800 nM of SRB; Dose 4: 40 nM of BCL, 40 nM of NTX and 4 μM of SRB), myelination was significantly improved (featured by an increased length of MBP-stained segments). **(G)** The synergistic potential of PXT3003 (Dose 2: 1.6 nM of BCL, 1.6 nM of NTX and 160 nM of SRB) was displayed by an isobologram plotted from dose-effect curves of each drug. The calculated Combination Index (CI) of synergy was 0.36; Synergism is characterized by a CI < 1. Greyed surface represents the concentrations of the mixes at which CI = 1. Three independent DRG co-cultures with 6 replicates each were performed and analysed. +, *P < 0.05; ++, **P < 0.01; ***P < 0.001 vs Vehicle; ANOVA with Dunnett’s test (*) and *t*-test (+). Data are shown as mean + SEM. Vhc: Vehicle.

Increased *PMP22* copy number in CMT1A patients causes peripheral nerve dysmyelination through dysbalance in the expression of genes for myelin proteins and consecutive downstream signalling events [3]. It can be studied in neuron-Schwann cell co-cultures derived from dorsal root ganglia (DRG) of CMT1A transgenic (TG) rats, a model of human CMT1A [8,40]. In this model, axons show reduced amounts of myelinated segments when compared to the wild-type [40]. We treated DRG co-cultures from CMT1A rats during 10–11 days with single drugs and their combination PXT3003. Myelination was measured by quantifying myelin basic protein (MBP)-stained segments of neuronal fibres (Figure 2A and B). We observed that, acting alone, each of the three drugs improved myelination in a dose-dependent fashion (Figure 2C to E). Then, we tested their combination PXT3003 and also demonstrated a dose-related effect on axonal myelination (Figure 2F) with a synergistic interaction between the 3 drugs (dose 2) as calculated through Combination Index analysis (CI = 0.36) and 3D isobologram obtained from dose-effect curves [23] (Figure 2G), supporting the concept of an increased drug potency when used in combination.

### PXT3003 modulates *Pmp22* mRNA levels *in vitro*

**Figure 3:**
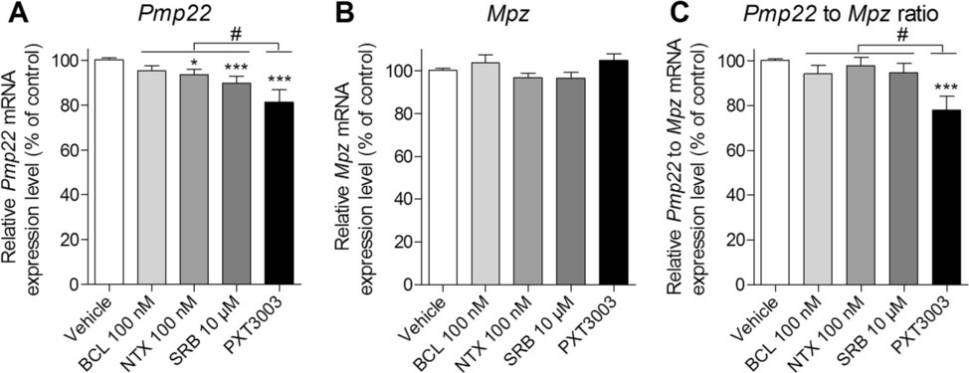
**BCL, NTX, SRB and their mix (PXT3003) down-regulate** ***Pmp22*** **mRNA expression in RT4 Schwannoma cells. (A)** After a treatment of 8 hours of RT4 Schwannoma cells with single compounds (100 nM of BCL, 100 nM of NTX or 10 μM of SRB) and with PXT3003 combination (100 nM of BCL, 100 nM of NTX and 10 μM of SRB; same drug ratio that was used for all doses in DRG co-cultures), a significant decrease of *Pmp22* mRNA expression level was observed when compared to vehicle (*Actb* and *Rps9* used together as reference genes) with a significant observed higher efficacy of PXT3003 over single compounds. **(B)** The same drugs had no effect on *Mpz* expression (*Actb* and *Rps9* used as reference genes). **(C)** The expression of *Pmp22* with respect to *Mpz* was significantly decreased after PXT3003 treatment, unlike single compounds. Three different experiments using 2 cultures each with 3 replicates were performed and analysed. *P < 0.05, ***P < 0.001 vs Vehicle; ANOVA with Dunnett’s test. ^#^ *P* < 0.05 vs the most active compound; post-hoc contrast test. Data are shown as mean + SEM.

We then tested the potency of the three drugs to down-regulate *Pmp22* expression in rat RT4 schwannoma when normalised to the levels of both *Actb* and *Rps9* housekeeping genes [41]. Using a drug ratio corresponding to the synergistic effect when combined in DRG co-cultures (Figure 2G, Dose 2), we observed moderately reduced levels of *Pmp22* transcript for single drug treatment with NTX or SRB (Figure 3A). Importantly, in the PXT3003 drug combination, this effect was increased, i.e. with significant difference to the single drug action (Figure 3A). We also found that *Mpz* mRNA, coding for the major protein of peripheral myelin, was not affected by our drugs (Figure 3B), singly or in combination, while the ratio of *Pmp22* to *Mpz* mRNA was significantly lowered (Figure 3C) by the drug combination but not by the single drugs. These results confirmed the importance of combining the chosen drugs and prompted us to use the PXT3003 combination in further testing in animal models.

### PXT3003 down-regulates *Pmp22* expression and is active in the *in vivo* transgenic rat model of CMT1A disease

**Figure 4:**
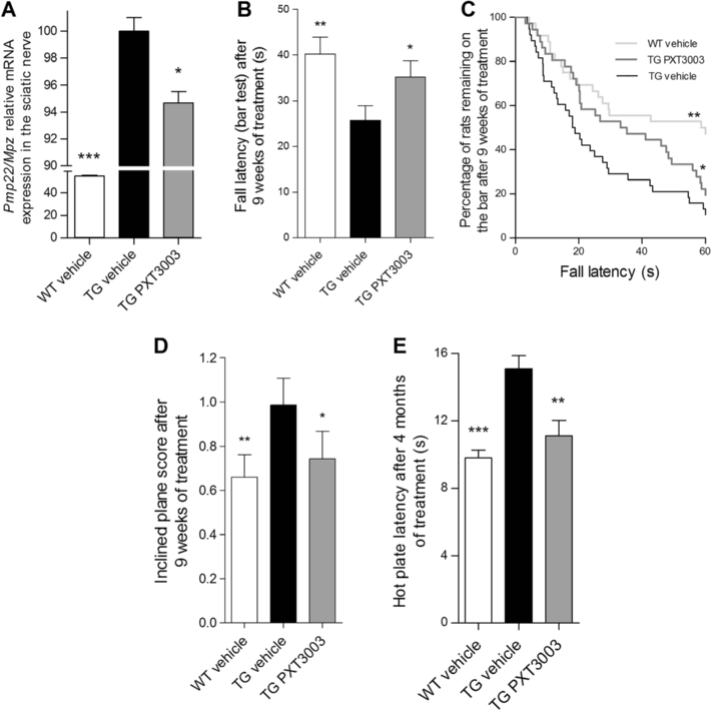
**Daily oral treatment of CMT1A rats with PXT3003 down-regulates** ***Pmp22*** **expression and reduces signs of motor and sensory neuropathy. (A)** A 9-week treatment with PXT3003 (BCL 30 μg/kg, NTX 3.5 μg/kg and SRB 1.05 mg/kg) decreased *Pmp22* to *Mpz* mRNA ratio in the sciatic nerve. *n* = 18, 20 and 18 animals for respectively WT vehicle, TG vehicle and TG PXT3003 groups. **(B)** Bar test. The latency to fall for transgenic rats was significantly improved after 9 weeks of treatment with PXT3003. **(C)** Kaplan-Meier representation of the data set in **(B)** demonstrated a positive effect of PXT3003. **(D)** After 9 weeks of treatment, the inclined plane score was significantly improved for transgenic rats. **(B)** to **(D)** *n* = 36, 38 and 36 animals for respectively WT vehicle, TG vehicle and TG PXT3003 groups. **(** **E** **)** A 4-month long PXT3003 treatment normalised the impaired thermal sensitivity of transgenic rats in the hot plate test. *n* = 12, 10 and 7 animals respectively for WT, vehicle, TG vehicle and TG PXT3003 groups. *P < 0.05; **P < 0.01; ***P < 0.001 vs TG vehicle; ANOVA with Dunnett’s test (except in **(C)**, logrank). Data are shown as mean + SEM.

PXT3003 was assessed in CMT1A rats [5,9]. These rats carry additional copies of the murine *Pmp22* gene leading to 1.6-fold overexpression of *Pmp22* mRNA in peripheral nerves. This model is characterised by dysmyelination, reduced MNCV, diminished muscle strength and sensory nerve involvement, mimicking the clinical phenotype of human CMT1A patients. CMT1A rats have been used previously to assess the efficacy of progesterone antagonists and recombinant Neuregulin 1 (NRG1) as single drugs [3,9,13,42]. Young adult male *Pmp22* TG rats and their WT littermates were treated during 9 weeks by daily oral treatment starting at 4 weeks of age. We confirmed by RTqPCR analysis the ~1.6-fold up-regulation of normalised *Pmp22* mRNA relative to *Mpz* mRNA in the TG group when compared to WT littermates (Figure 4A). In order to examine the ability of PXT3003 to lower *Pmp22* gene levels *in vivo*, we determined the expression of this gene in sciatic nerves at the end of 9 weeks of PXT3003 treatment. Importantly, we observed its significant but limited (−12%) down-regulation (Figure 4A) in line with the *in vitro* data obtained with Schwannoma cells (Figure 3C).

Efficacy of 9 weeks of oral daily PXT3003 treatment given to CMT1A TG rats was assessed by (i) measuring muscle strength in the bar test, (ii) performance on an inclined plane and (iii) thermal sensitivity in the hot plate test. We observed a substantial heterogeneity of TG rats with respect to muscular strength, as previously described [13,42]. After 9 weeks of treatment, the overall mean latency to fall (bar test) was significantly prolonged (Figure 4B). The same data set represented as Kaplan-Meier curves allowed us to confirm the significant improvement of PXT3003-treated animals (Figure 4C). It is important to note that the CMT1A-like phenotype was already manifested before the start of the treatment (Additional file 2A). We also assessed motor performance with the inclined plane test [14] that measures the ability of animals to rest on the plane fixed at two different angles. Before treatment, there was a substantial and significant difference between TG vehicle and WT groups in this test (Additional file 2B). After 9 weeks of treatment with PXT3003, CMT1A rats showed significantly improved performance on the inclined plane that even reached the level of performance of non-affected WT rats (Figure 4D).

CMT1A neuropathy is also characterised by sensory impairment [43], which we measured in the CMT1A rats using the hot plate [42]. We confirmed the reduced heat sensitivity reported previously in this model [42]. The daily treatment with PXT3003 over 4 months restored sensitivity of CMT1A rats almost to WT levels (Figure 4E).

### PXT3003 improves histological and electrophysiological parameters in CMT1A rats

**Figure 5:**
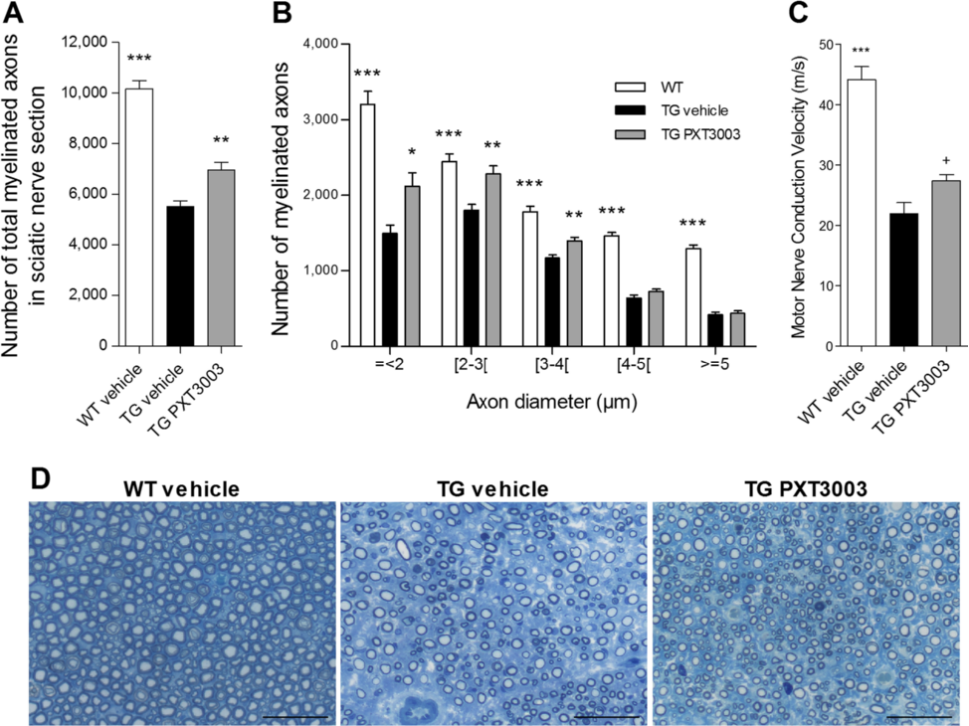
**Daily oral treatment of CMT1A rats with PXT3003 improves myelination and electrophysiology.** A 4-month PXT3003 treatment (BCL 30 μg/kg, NTX 3.5 μg/kg and SRB 1.05 mg/kg) of TG rats significantly increased the number of myelinated axons in sciatic nerve cross sections **(A)** mostly in the small to medium sized axon class of myelinated axons (< 4 μm) **(B)**. *n* = 12, 12 and 15 animals for respectively WT vehicle, TG vehicle and TG PXT3003 groups. **(C)** 8-month PXT3003 treatment increased the motor nerve conduction velocity of TG rats in sciatic nerve. *n* = 11, 9 and 7 animals for respectively WT vehicle, TG vehicle and TG PXT3003 groups. **(D)** Representative images of toluidine blue-stained sciatic nerve cross sections for each group. Scale bar: 25 μm. *,+ *P* < 0.05; ** *P* < 0.01; ***P < 0.001 vs TG vehicle; ANOVA with Dunnett’s test (except in **(C)**, + *t*-test). Data are shown as mean + SEM.

In order to confirm the clinical amelioration on the histological level, we examined sciatic nerve cross sections. In untreated transgenic rats at the age of 5 months, we observed a reduced number of myelinated fibres (Figure 5A), evident both for small (diameter < 4 μm) and large (diameter > 4 μm) axons (Figure 5B and D). Importantly, after 4 months of treatment with PXT3003, CMT1A TG rats exhibited a significant 31% increase in the number of myelinated fibres per nerve cross section (Figure 5A). This occurred mostly in small to middle-sized axons (Figure 5B and D). However, similarly to the effect of onapristone [13] in this model, PXT3003 failed to normalise another myelination parameter: the distribution of g-ratio as a function of axon diameter (Additional file 3A).

One consequence of dysmyelination in CMT1A disease is an impairment of electrophysiological parameters such as decrease of MNCV or of CMAP amplitude [43,44]. After 8 months of PXT3003 treatment in CMT1A rats, MNCV was improved (Figure 5C). However, CMAP amplitudes had only a slight statistical trend for improvement (Additional file 3B). All above treatment effects and durations in CMT1A rats are summarised in Additional file 4.

### PXT3003 improves axonal myelination and regeneration in a nerve crush model

**Figure 6:**
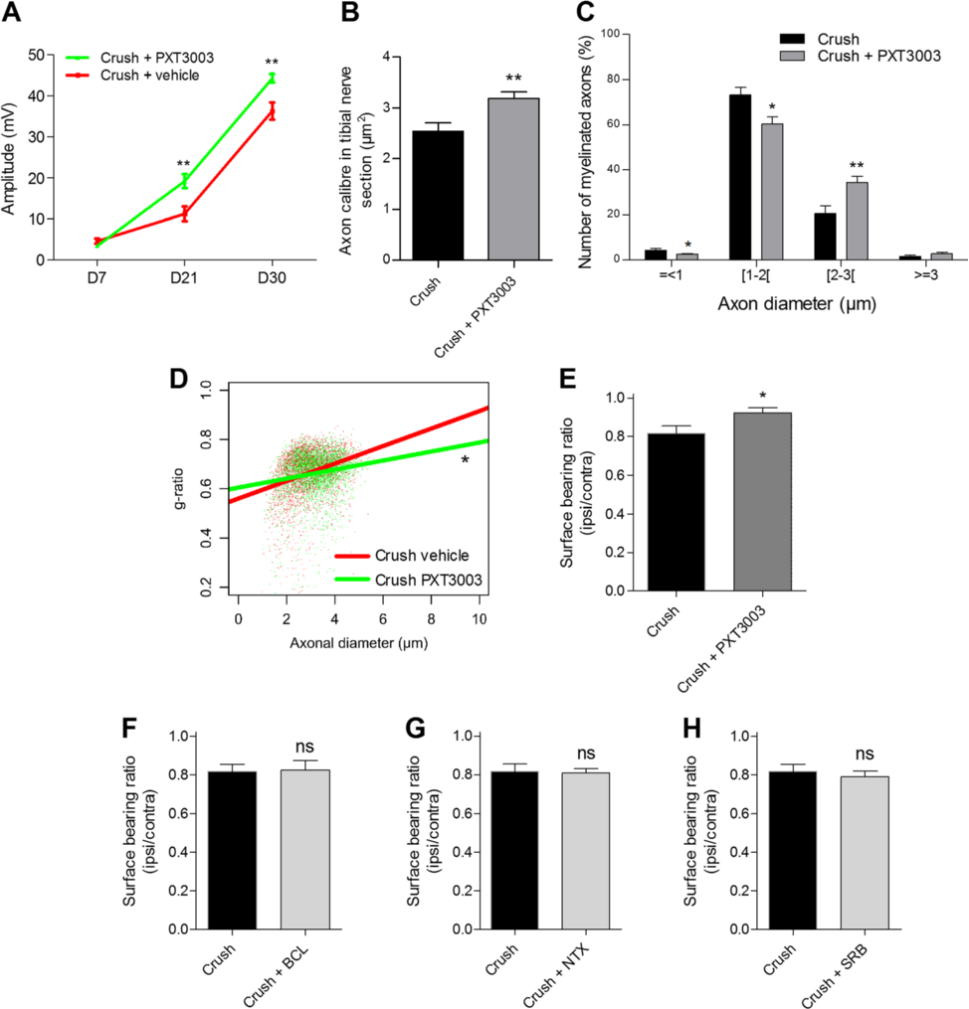
**Daily oral PXT3003 treatment of nerve-crushed mice restores nerve physiology, improves axonal and myelin integrity and functional behaviour. (A)** A 21 and 30 day-treatment with PXT3003 (BCL 60 μg/kg, NTX 7 μg/kg and SRB 2.1 mg/kg) increased the amplitude of CMAP (measured in the gastrocnemius) of crushed nerves in male mice. *n* = 10 animals in each group. **(B)** to **(D)** A 42 day-treatment with PXT3003 significantly improved axonal calibre size **(B)**, normalised the distribution of the number of myelinated axons in terms of axonal diameter **(C)** and improved the distribution of myelin g-ratio in tibial nerve cross sections **(D)**. *n* = 6 for each group. (**E**) After 13 days of treatment, PXT3003 (BCL 600 μg/kg, NTX 70 μg/kg and SRB 21 mg/kg) improved significantly the functional activity of crushed mice as assessed by the paw surface pressure against the floor (paw surface bearing) of the affected paw normalised to the contralateral non-affected one. **(F)** to **(H)** In contrast to PXT3003, single drugs had no effect on surface bearing ratio. *n* = 10 for each group. *P < 0.05, **P < 0.01, ***P < 0.001 vs Crush Vehicle; *t*–test. Data are shown as mean ± SEM.

To further assess the ability of PXT3003 to improve peripheral nerve function, we used the *in vivo* sciatic nerve crush model in mice. The nerve crush model is mostly considered as a model of Wallerian degeneration but demonstrates striking similarities to inherited demyelinating neuropathies with common features of axonal degeneration and dedifferentiation of Schwann cells [45]. In this model, nerve-crush damage results in temporal loss of function (lasting 30 days after nerve crush) accompanied by initial loss and gradual restoration of the amplitude of CMAP (Additional file 5A) as well as by the impairment of axonal morphological parameters (decrease of axonal calibre of the tibial nerve, impaired distribution of myelinated axons and of myelin g-ratio in crushed nerves) in regenerating nerves (Additional file 5B to D). When mice were treated once a day with PXT3003 starting 30 minutes after nerve crush, CMAP amplitudes (Figure 6A) measured at 21 and 30 days were largely normalised. At the end of a 42 day long drug treatment, axonal morphology (Figure 6B), distribution of myelinated fibres (Figure 6C), as well as the distribution g-ratio as an indicator of myelin thickness and/or of the axonal diameter (Figure 6D) were also positively affected in regenerating nerves.

Next, we tested the potency of the PXT3003 combination compared to single drugs in the functional behavioural assay of paw surface bearing, where the surface generated by paw pressure against the floor was measured [16]. In operated mice, unable to set down properly their affected paw to perform normal movements, a substantial decrease in the surface bearing of the affected hind paw was confirmed (Additional file 5E). After treatment of mice with PXT3003, using doses used in the experiments described above, no functional improvement was observed. However, when we increased drug doses to a level that was still less than the range of clinical use for their principal indications, i.e. 1/27 of the approved dosage for BCL (80 mg/day), 1/147 of approved NTX (50 mg/day) and 1/147 of approved SRB (15 g/day), PXT3003 was able to normalise completely this functional defect (Figure 6E) in contrast to single drugs that had no detectable effect (Figure 6F to H). These results highlight a neuro-regenerative and promyelinating potential of our drug combination.

We found no evidence of side-effects in any of the animal experiments described above (see Methods).

## Discussion

It is striking that a small change in the copy number of *PMP22* (three compared to two) is responsible for axonal loss in peripheral nerves, and consecutive muscle atrophy and impaired sensitivity in affected individuals. Functional integrity of myelin sheaths is highly sensitive to the stoichiometry of its components, and any dysfunction in the transcriptional network regulating expression of myelin genes is potentially deleterious. Systems biology analysis of available experimental data allowed us to prioritise an integral network of cellular signalling pathways implicated in transcriptional regulation of the *PMP22* gene. The converging point of this network is cAMP-dependent silencer upstream of the *PMP22* promoter. Three drugs: baclofen, naltrexone and sorbitol, all binding to GPCR receptors and affecting intracellular cAMP levels, were chosen among available authorised drugs with additional criteria of safety and ability to affect other aspects of nerve function. The underlying idea was that their combined action will be more potent which could permit their use at lower doses further minimising potential unwanted secondary effects. Other myelin genes can be potentially down-regulated by these drugs, which necessitated an experimental verification of our underlying hypothesis.

We performed this primary analysis by testing the three molecules in a disease-relevant *ex vivo* myelination model derived from *Pmp22* transgenic rats. While separately the three molecules were able to increase myelination, their common effect demonstrated a positive synergistic interaction that provided a rationale for further combination assays. In pilot experiments using cultured Schwannoma cells, we were able to detect a limited but significant down-regulation of *Pmp22* gene expression. The RT4-D6P2T Schwannoma cell line that we used displayed features that resemble those of normal myelinating Schwann cells with the expression of myelin genes at relatively high levels [46,47]. The extent of the down-regulation observed was significantly higher for the combination compared to the action of individual drugs. Expression of the major myelin gene *Mpz* was not affected by these treatments, while *Pmp22* gene expression (when normalised to that of *Mpz*) was decreased by the presence of PXT3003 but not by its constitutive drugs. Interestingly, this specific effect on *Pmp22* but not on *Mpz* expression is similar to the effect observed for the progesterone antagonist onapristone [9]. We note that CMT1A is a gene dosage disease where translation, processing and transport of PMP22 protein in Schwann cells is not yet fully elucidated and only a small fraction of the protein is incorporated into mature myelin [5]. Moreover, PMP22 protein and mRNA expression in CMT1A patients is variable and no correlation was found between PMP22 overexpression and the Charcot-Marie-Tooth disease neuropathy score (CMTNS) clinical score [42,48,49]. Future studies may shed light on the effect of PXT3003 on the regulation of PMP22 protein and other myelin proteins in peripheral tissue of CMT1A patients.

Since PXT3003 proved efficacy over its single compounds in the cellular models, we further tested the combination drug in the transgenic rat model of CMT1A. As patients included in clinical trials could have manifested CMT1A for years before the beginning of the trial, we used CMT1A adult rats for PXT3003 treatment. We also asked whether electrophysiological amelioration could be expected after a long time of treatment, i.e. 8 months, when the disease has reached an advanced stage in the animal model. Here, we demonstrated the beneficial effect of PXT3003 on the clinically relevant muscle motor performance and sensory nerve function. Several biochemical, electrophysiological and morphological characteristics such as the ratio of *Pmp22/Mpz* gene expression in sciatic nerves, small fibre myelination and MNCV were significantly improved by PXT3003. Of note, the mild reduction in *Pmp22* expression after PXT3003 treatment (15%) is reminiscent of the therapeutic effect of onapristone in CMT1A rats [9]. We here argue that the reduction of *Pmp22* by PXT3003 improves peripheral nerve myelination and/or trophic support to axons leading to the increase of the number of myelinated axons in the sciatic nerve by 31%. At the same time, PXT3003 combination had limited effect on CMAP and myelin g-ratio in this CMT1A TG rat model in contrast to the acute *in vivo* nerve crush model. Further work will be needed to see if this increase in NCV without changes in myelin g-ratio may be due to the changes in internodal lengths. We note that the speed of nerve impulse propagation is also determined by the internodal length, which could explain ameliorated NCV and unaltered CMAP. In addition, we hypothesise that the absence of the effect on CMAP at this moment may be due to the fact that the treatment of TG rats had been started late, in early adulthood when the disease was already at an advanced, chronic stage. We note that in the progesterone antagonist therapy trial, a stronger therapeutic effect was observed when treatment was initiated early postnatally [9,13]. Also, early postnatal treatment of CMT1A rats with soluble NRG corrected the dysmyelinating and dedifferentiated Schwann cell phenotype [3]. Experiments with an early onset treatment paradigm with PXT3003 in CMT1A rats are in progress and will also focus on its effects on the differentiation state of *Pmp22* transgenic Schwann cells. Finally, we note that in the nerve crush axonal regeneration model, PXT3003 administrated very early, i.e. 30 minutes after nerve crush, accelerated the process of axonal regeneration thus leading to the significantly decreased proportion of small myelinated axons and causing improvement in the amplitude of CMAP. These observations might also raise the possibility that PXT3003 could modify directly axons activity enabling their protection or regeneration, in agreement with our initial hypothesis (Figure 1B).

The synergy observed in models permits the use of individual drugs at lower doses up to several hundred times less than what is being used in their primary indications, diminishing potential undesirable secondary effects.

Throughout this study, we focused on the validation of the efficacy of PXT3003 in cellular and animal models by assessing several CMT1A disease-relevant endpoints. Our goal was more on the translational side to provide the rationale for further testing in an affected human population. The major point of this work was to provide a proof of principle for the repurposing of approved and clinically safe drugs, using reproducible preclinical systems with the main accent to genetic disease model. Many features of CMT1A disease are far from being understood, neither in rats nor in human patients. It is important to keep in mind that, although for some endpoints the effect in some models was significant but rather limited, it can be considered as successful test of the hypothesis that approved drugs can be newly formulated to ameliorate a disease that they were not designed for.

Although designed from a series of mechanistic hypotheses, our preclinical data remains for the moment mostly observational. More efforts must be performed for a better understanding of exact mechanism of action and the combinational synergy observed for PXT3003. It will be particularly relevant to verify experimentally the involvement of GABA_B_, opioid or muscarinic receptors in the therapeutic effect reported here. Investigating the mechanism of synergy could be even more challenging. Launching these sophisticated and laborious mechanistic experiments was out of scope of this study also because of limited correlations generally observed for efficacy when translating data in animal models to the human clinical studies [50]. Finally, as the profile of PXT3003 seemed rather safe, taking into account the nature and low doses of the 3 compounds, we decided to concentrate our efforts on its testing in an exploratory phase 2. This has recently provided first indications of therapeutic activity in mild to moderately affected adult CMT1A patients [51]. Altogether these data indicate that PXT3003 deserves further clinical investigation as well as experimental elucidation of its detailed mechanism of action.

## Conclusions

In conclusion, the novel combination of three well-known and approved drugs (baclofen, naltrexone and sorbitol), identified by our Systems Biology approach, is able to improve myelination in the *ex vivo* myelination model of CMT1A, while single drugs displayed a lower efficacy. The combination is also able to down-regulate the expression of *Pmp22* in cultured Schwannoma cells more efficiently than single drugs. The tests performed *in vivo* to assess the efficacy of the combination in the nerve crush mouse model demonstrate the acute neuro-regenerative and promyelinating potential of PXT3003 with the functional test demonstrating superiority of the combined action. Moreover, PXT3003 combination improves relevant parameters in the CMT1A rat model (muscular performances, heat sensitivity, histology and electrophysiology). No toxic effect of PXT3003 has been detected in animal studies.

Rational polytherapy based on a combinational repositioning of existing non-toxic drugs that act on pleiotropic pathways, may represent an important novel approach for rapid drug development in a variety of disorders. Future work will show if a similar approach could be useful in the treatment of other neglected orphan diseases where therapies are urgently needed.

## Additional files

Additional file 1:
**Pharmacology-network based drug repurposing for CMT1A disease.**
Additional file 2:
**Motor performances are significantly affected in CMT1A rats.**
Additional file 3:
**Myelination and CMAP are unchanged after PXT3003 treatment.**
Additional file 4:
**Summary of treatment characteristics in CMT1A rats.**
Additional file 5:
**Nerve electrophysiology, myelin integrity and bearing function are substantially affected by the sciatic nerve crush.**

## References

[1] Patzkó A, Shy ME (2011). Update on Charcot-Marie-Tooth disease. Curr Neurol Neurosci Rep.

[2] Rossor AM, Polke JM, Houlden H, Reilly MM (2013). Clinical implications of genetic advances in Charcot-Marie-Tooth disease. Nat Rev Neurol.

[3] Fledrich R, Stassart RM, Klink A, Rasch LM, Prukop T, Haag L, Czesnik D, Kungl T, Abdelaal TAM, Keric N, Stadelmann C, Brück W, Nave K-A, Sereda MW (2014). Soluble neuregulin-1 modulates disease pathogenesis in rodent models of Charcot-Marie-Tooth disease 1A. Nat Med.

[4] Nave K-A, Trapp BD (2008). Axon-glial signaling and the glial support of axon function. Annu Rev Neurosci.

[5] Sereda M, Griffiths I, Pühlhofer A, Stewart H, Rossner MJ, Zimmerman F, Magyar JP, Schneider A, Hund E, Meinck HM, Suter U, Nave KA (1996). A transgenic rat model of Charcot-Marie-Tooth disease. Neuron.

[6] Cosgaya JM, Chan JR, Shooter EM (2002). The neurotrophin receptor p75NTR as a positive modulator of myelination. Science.

[7] Rangaraju S, Madorsky I, Pileggi JG, Kamal A, Notterpek L (2008). Pharmacological induction of the heat shock response improves myelination in a neuropathic model. Neurobiol Dis.

[8] Callizot N, Combes M, Steinschneider R, Poindron P (2011). A new long term in vitro model of myelination. Exp Cell Res.

[9] Sereda MW, Meyer zu Hörste G, Suter U, Uzma N, Nave K-A (2003). Therapeutic administration of progesterone antagonist in a model of Charcot-Marie-Tooth disease (CMT-1A). Nat Med.

[10] Tashiro H, Fukuda Y, Kimura A, Hoshino S, Ito H, Dohi K (1996). Assessment of microchimerism in rat liver transplantation by polymerase chain reaction. Hepatology.

[11] Hirst JA, Howick J, Aronson JK, Roberts N, Perera R, Koshiaris C, Heneghan C (2014). The need for randomization in animal trials: an overview of systematic reviews. PLoS One.

[12] Kilkenny C, Browne WJ, Cuthill IC, Emerson M, Altman DG (2010). Improving bioscience research reporting: the ARRIVE guidelines for reporting animal research. J Pharmacol Pharmacother.

[13] Meyer Zu Horste G, Prukop T, Liebetanz D, Mobius W, Nave K-A, Sereda MW (2007). Antiprogesterone therapy uncouples axonal loss from demyelination in a transgenic rat model of CMT1A neuropathy. Ann Neurol.

[14] Rivlin AS, Tator CH (1977). Objective clinical assessment of motor function after experimental spinal cord injury in the rat. J Neurosurg.

[15] Beyreuther BK, Callizot N, Brot MD, Feldman R, Bain SC, Stöhr T (2007). Antinociceptive efficacy of lacosamide in rat models for tumor- and chemotherapy-induced cancer pain. Eur J Pharmacol.

[16] Tétreault P, Dansereau M-A, Doré-Savard L, Beaudet N, Sarret P (2011). Weight bearing evaluation in inflammatory, neuropathic and cancer chronic pain in freely moving rats. Physiol Behav.

[17] Bordet T, Buisson B, Michaud M, Drouot C, Gale P, Delaage P, Akentieva NP, Evers AS, Covey DF, Ostuni MA, Lacape J, Massaad C, Schumacher M, Steidl E, Maux D, Delaage M, Henderson CE, Pruss RM (2007). Identification and characterization of cholest-4-en-3-one, oxime (TRO19622), a novel drug candidate for amyotrophic lateral sclerosis. J Pharmacol Exp Ther.

[18] Markowski C, Markowski E (1990). Conditions for the effectiveness of a preliminary test of variance. Am Stat.

[19] Sawilowsky SS, Blair RC (1992). A more realistic look at the robustness and Type II error properties of the t test to departures from population normality. Psychol Bull.

[20] Grabovsky Y, Tallarida RJ (2004). Isobolographic analysis for combinations of a full and partial agonist: curved isoboles. J Pharmacol Exp Ther.

[21] Chou T-C (2006). Theoretical basis, experimental design, and computerized simulation of synergism and antagonism in drug combination studies. Pharmacol Rev.

[22] Geary N (2013). Understanding synergy. Am J Physiol Metab.

[23] Tallarida RJ (2006). An overview of drug combination analysis with isobolograms. J Pharmacol Exp Ther.

[24] Suter U, Snipes GJ, Schoener-Scott R, Welcher AA, Pareek S, Lupski JR, Murphy RA, Shooter EM, Patel PI (1994). Regulation of tissue-specific expression of alternative peripheral myelin protein-22 (PMP22) gene transcripts by two promoters. J Biol Chem.

[25] Sabéran-Djoneidi D, Sanguedolce V, Assouline Z, Lévy N, Passage E, Fontés M (2000). Molecular dissection of the Schwann cell specific promoter of the PMP22 gene. Gene.

[26] Ogata T, Iijima S, Hoshikawa S, Miura T, Yamamoto S, Oda H, Nakamura K, Tanaka S (2004). Opposing extracellular signal-regulated kinase and Akt pathways control Schwann cell myelination. J Neurosci.

[27] Arthur-Farraj P, Wanek K, Hantke J, Davis CM, Jayakar A, Parkinson DB, Mirsky R, Jessen KR (2011). Mouse schwann cells need both NRG1 and cyclic AMP to myelinate. Glia.

[28] Pereira JA, Lebrun-Julien F, Suter U (2012). Molecular mechanisms regulating myelination in the peripheral nervous system. Trends Neurosci.

[29] Faroni A, Magnaghi V (2011). The neurosteroid allopregnanolone modulates specific functions in central and peripheral glial cells. Front Endocrinol (Lausanne).

[30] Procacci P, Ballabio M, Castelnovo LF, Mantovani C, Magnaghi V (2012). GABA-B receptors in the PNS have a role in Schwann cells differentiation?. Front Cell Neurosci.

[31] Glenn TD, Talbot WS (2013). Signals regulating myelination in peripheral nerves and the Schwann cell response to injury. Curr Opin Neurobiol.

[32] Towers S, Princivalle A, Billinton A, Edmunds M, Bettler B, Urban L, Bowery NG (2000). GABA B receptor protein and mRNA distribution in rat spinal cord and dorsal root ganglia. Eur J Neurosci.

[33] Wang H-Y, Frankfurt M, Burns LH (2008). High-affinity naloxone binding to filamin a prevents mu opioid receptor-Gs coupling underlying opioid tolerance and dependence. PLoS One.

[34] Hytrek SD, McLaughlin PJ, Lang CM, Zagon IS (1996). Inhibition of human colon cancer by intermittent opioid receptor blockade with naltrexone. Cancer Lett.

[35] Zhu M, Li RC (1999). Receptor binding activities of Schefflera triterpenoids and oligosaccharides. Planta Med.

[36] Singer MA, Lindquist S (1998). Multiple effects of trehalose on protein folding in vitro and in vivo. Mol Cell.

[37] Kumar R (2009). Role of naturally occurring osmolytes in protein folding and stability. Arch Biochem Biophys.

[38] Notterpek L, Ryan MC, Tobler AR, Shooter EM (1999). PMP22 accumulation in aggresomes: implications for CMT1A pathology. Neurobiol Dis.

[39] Schlebach JP, Peng D, Kroncke BM, Mittendorf KF, Narayan M, Carter BD, Sanders CR (2013). Reversible folding of human peripheral myelin protein 22, a tetraspan membrane protein. Biochemistry.

[40] Nobbio L, Mancardi G, Grandis M, Levi G, Suter U, Nave KA, Windebank AJ, Abbruzzese M, Schenone A (2001). PMP22 transgenic dorsal root ganglia cultures show myelin abnormalities similar to those of human CMT1A. Ann Neurol.

[41] Vandesompele J, De Preter K, Pattyn F, Poppe B, Van Roy N, De Paepe A, Speleman F (2002). Accurate normalization of real-time quantitative RT-PCR data by geometric averaging of multiple internal control genes. Genome Biol.

[42] Fledrich R, Schlotter-Weigel B, Schnizer TJ, Wichert SP, Stassart RM, Meyer zu Hörste G, Klink A, Weiss BG, Haag U, Walter MC, Rautenstrauss B, Paulus W, Rossner MJ, Sereda MW (2012). A rat model of Charcot-Marie-Tooth disease 1A recapitulates disease variability and supplies biomarkers of axonal loss in patients. Brain.

[43] Shy ME, Chen L, Swan ER, Taube R, Krajewski KM, Herrmann D, Lewis RA, McDermott MP (2008). Neuropathy progression in Charcot-Marie-Tooth disease type 1A. Neurology.

[44] Pareyson D, Scaioli V, Laurà M (2006). Clinical and electrophysiological aspects of Charcot-Marie-Tooth disease. Neuromolecular Med.

[45] Martini R, Klein D, Groh J (2013). Similarities between inherited demyelinating neuropathies and Wallerian degeneration: an old repair program may cause myelin and axon perturbation under nonlesion conditions. Am J Pathol.

[46] Castorina A, Scuderi S, D’Amico AG, Drago F, D’Agata V (2014). PACAP and VIP increase the expression of myelin-related proteins in rat schwannoma cells: involvement of PAC1/VPAC2 receptor-mediated activation of PI3K/Akt signaling pathways. Exp Cell Res.

[47] Hai M, Muja N, DeVries GH, Quarles RH, Patel PI (2002). Comparative analysis of Schwann cell lines as model systems for myelin gene transcription studies. J Neurosci Res.

[48] Nobbio L, Visigalli D, Radice D, Fiorina E, Solari A, Lauria G, Reilly MM, Santoro L, Schenone A, Pareyson D (2014). PMP22 messenger RNA levels in skin biopsies: testing the effectiveness of a Charcot-Marie-Tooth 1A biomarker. Brain.

[49] Katona I, Wu X, Feely SME, Sottile S, Siskind CE, Miller LJ, Shy ME, Li J (2009). PMP22 expression in dermal nerve myelin from patients with CMT1A. Brain.

[50] Hay M, Thomas DW, Craighead JL, Economides C, Rosenthal J (2014). Clinical development success rates for investigational drugs. Nat Biotechnol.

[51] Attarian S, Vallat J-M, Magy L, Funalot B, Gonnaud P-M, Lacour A, Péréon Y, Dubourg O, Pouget J, Micallef J, Franques J, Lefèvre M-N, Ghorab K, Al-Moussawi M, Tiffreau V, Magot A, Leclair-Visonneau L, Stojkovic T, Bossi L, Lehert P, Walter G, Bertrand V, Mandel J, Milet A, Hajj R, Boudiaf L, Scart-Grès C, Nabirotchkin S, Guedj M, Chumakov I, *et al*: **An Exploratory randomised double-blind and placebo-controlled phase 2 study of a combination of baclofen, naltrexone and sorbitol (PXT3003) in patients with Charcot-Marie-Tooth disease type 1A.** *Orphanet J Rare Dis* 2014, **9:**199. 10.1186/s13023-014-0199-0 PMC4311411 25519680

