# Supplementary material for: Polytherapy with a combination of three repurposed drugs (PXT3003) down-regulates Pmp22 over-expression and improves myelination, axonal and functional parameters in models of CMT1A neuropathy
Source: Orphanet J Rare Dis. 2014 Dec 10;9:201. doi: 10.1186/s13023-014-0201-x (PMC4279797; doi:10.1186/s13023-014-0201-x)
Supplement: Additional file 1: — Pharmacology-network based drug repurposing for CMT1A disease. [file 13023_2014_201_MOESM1_ESM.pdf]

Additional file 1

Supporting text for Figure 1. Pharmacology-network based drug repurposing for CMT1A disease.

(A) Three principal pathways regulating expression of *PMP22* gene through extracellular GPCR signalling in Schwann cells. The presumed crosstalk between cAMP pathway, neurosteroid-mediated signalling and mutually-balanced PI3K-AKT/ERK kinase cascades provides functional signals regulating the expression of *PMP22* gene. cAMP signalling is implicated in different aspects of Schwann cell biology and influences their differentiation and myelin formation [1, 2]. cAMP/PKA module is able to modify the activity of several transcriptional factors implicated in the transcriptional control of *PMP22* gene. In cell cultures, cAMP increases the expression of PMP22 protein acting presumably, through down-regulation of inhibitory effect of cAMP-dependent silencer element in the promoter region of *PMP22* gene [3, 4]. The physical contact of Schwann cells with neurons is thought to control the intracellular levels of cAMP, modifying Schwann cell response to growth factors [1]. Moreover, the differentiated state of Schwann cells depends on the counter-balanced activation of ERK and PI3K-AKT pathways by growth factors, mediated through receptor-tyrosine kinases, with PI3K-AKT signalling promoting differentiation and expression of myelin proteins [5, 6]. It was shown that canonical cAMP-PKA-CREB pathway synergistically and in a dose-dependent manner enhances pro-myelination effects of NRG1 signalling likely mediated by AKT kinase and EGR2 transcription factor, a positive regulator of myelination program and *PMP22* transcription in Schwann cells [7, 8].

Neurosteroid progesterone and its derivatives (DHP and allopregnanolone) play an important role in myelin formation acting as autocrine regulatory factors. Transcriptional up-regulation of *PMP22* gene by the neurosteroids can be mediated or modified by cAMP signalling; thus,

treatment with allopregnanolone, a positive allosteric modulator of $GABA_A$ receptors, increases the level of intracellular cAMP and CREB phosphorylation in Schwann cells [4, 9–11]. We expected that these convergent signalling pathways in Schwann cells can be simultaneously regulated by different G-protein coupled receptors (GPCRs) either directly or via crosstalk with receptor tyrosine kinases, as it was shown in other cellular settings [12, 13]. Accordingly, pharmacological modulation of the G-protein coupled receptors opens the possibility for developing robust and safe combinational therapeutics decreasing the deleterious excessive expression of PMP22 protein and restoring the differentiation program in CMT1A Schwann cells.

GABA(B)R: metabotropic GABA receptor; OPRs: opioid receptors; FLNA: filamin A, alpha; POMC: proopiomelanocortin; PENK: proenkephalin; PDYN: prodynorphin; CHRMs: muscarinic receptors; Gαi: inhibitory subunit of G alpha proteins; Gαs: stimulatory subunit of G alpha proteins; ADCY: adenylate cyclase; PKA: cAMP-dependent protein kinase A; CREB: cAMP responsive element binding protein; NFKB: nuclear factor-kappa B; RTK: receptor tyrosine kinases; PI3K: phosphatidylinositol-4,5-bisphosphate 3-kinase; ERK: mitogen-activated protein kinase 1 and 3; AKT: v-akt murine thymoma viral oncogene homolog 1; GABA(A)R: inotropic GABA receptors; PR: nuclear progesterone receptor; EGR2: early growth response 2 transcription factor; PROG: progesterone, DHP: dihydroprogesterone; THP: allopregnanolone, positive modulator of GABA(A)R receptors. PROG, DHP and THP are neurosteroids produced by Schwann cells. cAMP: cyclic AMP; PDGF: platelet-derived growth factor; IGF1: insulin-like growth factor 1; NRG1: neuregulin 1; “silencer”: putative cAMP-dependent regulatory region in the promoter of *PMP22* gene.

(B) Cytoprotective and neuromodulator actions of PXT3003 drug combination in peripheral neurons. The reciprocal interactions of neuronal and Schwann cells assure correct processing of sensory and locomotor information in the peripheral nervous system (PNS) [14,

15]. We supposed that primarily functional abnormalities, induced by PMP22 overexpression in CMT1A Schwann cells, provoke a cascade of pathophysiological alterations in neurons [16]. Therefore, as an additional selection criterion for clinical development, we evaluated the potential capacity of candidate drugs for attenuating these secondary destructive effects in neuronal cells.

Schwann cells regulate the level of several neurotransmitters (GABA, ATP and glutamate) and inflammatory proteins in PNS [17–24]. Glutamate and ATP play an important role as excitatory and cytotoxic neurotransmitters under pathological conditions and are implicated in perturbed nociceptive signalling associated with inflammatory and neuropathic pain [25–33]. Dysfunction of CMT1A Schwann cells can not only disturb myelination process, but probably also affect levels of these neuromodulator substances, which can significantly compromise functional performance of neuronal signalling. Several publications demonstrated the excessive activation P2RX7 receptors, which modulate processing and release of CNTF and IL1B, in CMT1A Schwann cells [19, 34]. We hypothesized that increased neuronal excitability of sensory and motor neurons is responsible for development of at least some of pathological manifestations of CMT1A and represents an important functional target for therapeutic intervention in Charcot-Marie-Tooth disease.

Both $GABA_B$ and opioid receptors are powerful modulators of neuronal excitability and painful sensation [35–38]. $GABA_B$ receptors agonized by baclofen are able to inhibit P2X3 receptor-mediated neuronal excitability of nociceptive neurons, and attenuate NMDA-activated current in the primary sensory neurons [39, 40]. Basic molecular mechanism underlying antinociceptive effects of $GABA_B$ and opioid receptors can be mediated by the coupling of both receptors to activation G protein-gated inwardly rectifying K+ (GIRK) channels and inhibition of voltage-gated Ca^{2+} channels, though these metabotropic receptors are able also to modulate activity of TRPV1, voltage-gated sodium, ASICs and NMDA

receptors [38, 41–45]. Although not considered a significant symptom, pain is frequently complained by CMT1A patients [46, 47]. We expect that PXT3003 combination could attenuate sensory impairments accompanied development of Charcot-Marie-Tooth disease. $GABA_B$ and opioid receptors are not only potent modulators of neuronal excitability, but are also able to activate several cytoprotective signalling pathways in different experimental settings (some of the established signalling modules, implicated in the anti-apoptotic effect of $GABA_B$ and opioid receptors, are shown) [35, 45, 48–51]. For instance, both $GABA_B$ and opioid receptors can protect neuronal cells from apoptosis by transactivation of IGF-1R receptor [52–55]. Importantly, neurotropic insulin-like growth factor-1 (IGF-1) not only protects cell from various cytotoxic insults, but also promotes axonal growth from dorsal root ganglion (DRG) neurons [56].

Finally, muscarinic receptors, that might mediate therapeutic effect of sorbitol, are also recognized as important functional receptors in PNS, modulate neuronal activity of primary sensory neurons, are implicated in nociceptive sensation and could provide substantial neuroprotection from broad spectrum of cytotoxic factors [49, 57–59].

We suppose that PXT3003 combination could preserve functional integrity of neuronal cells in CMT patients by attenuating excessive excitability of peripheral neurons, normalize propagation of neuronal impulses and reduce axonal loss and functional perturbations at neuro-muscular junctions.

IGFR1: insulin-like growth factor 1 receptor; PKC: protein kinase C; SRC: v-src avian sarcoma (Schmidt-Ruppin A-2) viral oncogene homolog; AMPK: AMP-activated kinase; FAK: Focal adhesion kinase; BCL2: B-cell CLL/lymphoma 2; CACNAs: calcium channels, voltage-dependent; GRINs: ionotropic NMDA glutamate receptors; VGSC: sodium channels, voltage-gated; TRPV1: transient receptor potential cation channel, subfamily V, member 1;

ASICs: acid-sensing (proton-gated) ion channels; P2X3: purinergic receptor P2X, ligand-gated ion channel, 3; GIRKs: G protein-coupled inwardly-rectifying potassium channels.

Additional References

1. Morgan L, Jessen KR, Mirsky R: **The effects of cAMP on differentiation of cultured Schwann cells: progression from an early phenotype (04+) to a myelin phenotype (P0+, GFAP-, N-CAM-, NGF-receptor-) depends on growth inhibition.** *J Cell Biol* 1991, **112**:457–67.

2. Yoon C, Korade Z, Carter BD: **Protein kinase A-induced phosphorylation of the p65 subunit of nuclear factor-kappaB promotes Schwann cell differentiation into a myelinating phenotype.** *J Neurosci* 2008, **28**:3738–46.

3. Suter U, Snipes GJ, Schoener-Scott R, Welcher a a, Pareek S, Lupski JR, Murphy R a, Shooter EM, Patel PI: **Regulation of tissue-specific expression of alternative peripheral myelin protein-22 (PMP22) gene transcripts by two promoters.** *J Biol Chem* 1994, **269**:25795–808.

4. Sabéran-Djoneidi D, Sanguedolce V, Assouline Z, Lévy N, Passage E, Fontés M: **Molecular dissection of the Schwann cell specific promoter of the PMP22 gene.** *Gene* 2000, **248**:223–31.

5. Nave K-A, Salzer JL: **Axonal regulation of myelination by neuregulin 1.** *Curr Opin Neurobiol* 2006, **16**:492–500.

6. Ogata T, Iijima S, Hoshikawa S, Miura T, Yamamoto S, Oda H, Nakamura K, Tanaka S: **Opposing extracellular signal-regulated kinase and Akt pathways control Schwann cell myelination.** *J Neurosci* 2004, **24**:6724–32.

7. Taveggia C, Zanazzi G, Petrylak A, Yano H, Rosenbluth J, Einheber S, Xu X, Esper RM, Loeb J a, Shrager P, Chao M V, Falls DL, Role L, Salzer JL: **Neuregulin-1 type III determines the ensheathment fate of axons.** *Neuron* 2005, **47**:681–94.

8. Arthur-Farraj P, Wanek K, Hantke J, Davis CM, Jayakar A, Parkinson DB, Mirsky R, Jessen KR: **Mouse schwann cells need both NRG1 and cyclic AMP to myelinate.** *Glia* 2011, **59**:720–33.

9. Désarnaud F, Bidichandani S, Patel PI, Baulieu EE, Schumacher M: **Glucocorticosteroids stimulate the activity of the promoters of peripheral myelin protein-22 and protein zero genes in Schwann cells.** *Brain Res* 2000, **865**:12–6.

10. Guennoun R, Benmessahel Y, Delespierre B, Gouézou M, Rajkowski KM, Baulieu EE, Schumacher M: **Progesterone stimulates Krox-20 gene expression in Schwann cells.** *Brain Res Mol Brain Res* 2001, **90**:75–82.

11. Faroni A, Magnaghi V: **The neurosteroid allopregnanolone modulates specific functions in central and peripheral glial cells.** *Front Endocrinol (Lausanne)* 2011, **2**:103.

12. Shah BH, Catt KJ: **GPCR-mediated transactivation of RTKs in the CNS: mechanisms and consequences.** *Trends Neurosci* 2004, **27**:48–53.

13. Hagan CR, Daniel AR, Dressing GE, Lange C a: **Role of phosphorylation in progesterone receptor signaling and specificity.** *Mol Cell Endocrinol* 2012, **357**:43–9.

14. Nave K-A: **Myelination and support of axonal integrity by glia.** *Nature* 2010, **468**:244–52.

15. Nave K-A, Trapp BD: **Axon-glial signaling and the glial support of axon function.** *Annu Rev Neurosci* 2008, **31**:535–61.

16. Nave K-A, Sereda MW, Ehrenreich H: **Mechanisms of disease: inherited demyelinating neuropathies--from basic to clinical research.** *Nat Clin Pract Neurol* 2007, **3**:453–64.

17. Jeftinija SD, Jeftinija K V: **ATP stimulates release of excitatory amino acids from cultured Schwann cells.** *Neuroscience* 1998, **82**:927–34.

18. Tofaris GK, Patterson PH, Jessen KR, Mirsky R: **Denervated Schwann cells attract macrophages by secretion of leukemia inhibitory factor (LIF) and monocyte chemoattractant protein-1 in a process regulated by interleukin-6 and LIF.** *J Neurosci* 2002, **22**:6696–703.

19. Colomar A, Marty V, Médina C, Combe C, Parnet P, Amédée T: **Maturation and release of interleukin-1beta by lipopolysaccharide-primed mouse Schwann cells require the stimulation of P2X7 receptors.** *J Biol Chem* 2003, **278**:30732–40.

20. Liu GJ, Bennett MR: **ATP secretion from nerve trunks and Schwann cells mediated by glutamate.** *Neuroreport* 2003, **14**:2079–83.

21. Wu S-Z, Jiang S, Sims TJ, Barger SW: **Schwann cells exhibit excitotoxicity consistent with release of NMDA receptor agonists.** *J Neurosci Res* 2005, **79**:638–43.

22. Magnaghi V, Parducz A, Frasca A, Ballabio M, Procacci P, Racagni G, Bonanno G, Fumagalli F: **GABA synthesis in Schwann cells is induced by the neuroactive steroid allopregnanolone.** *J Neurochem* 2010, **112**:980–90.

23. Perego C, Di Cairano ES, Ballabio M, Magnaghi V: **Neurosteroid allopregnanolone regulates EAAC1-mediated glutamate uptake and triggers actin changes in Schwann cells.** *J Cell Physiol* 2012, **227**:1740–51.

24. Parpura V, Liu F, Jeftinija K V, Haydon PG, Jeftinija SD: **Neuroligand-evoked calcium-dependent release of excitatory amino acids from Schwann cells.** *J Neurosci* 1995, **15**:5831–9.

25. Hugon J, Vallat JM, Leboutet MJ: **Cytotoxic properties of glutamate and aspartate in rat peripheral nerves: histological findings.** *Neurosci Lett* 1987, **81**:1–6.

26. Jarvis MF: **Contributions of P2X3 homomeric and heteromeric channels to acute and chronic pain.** *Expert Opin Ther Targets* 2003, **7**:513–22.

27. McGaraughty S, Wismer CT, Zhu CZ, Mikusa J, Honore P, Chu KL, Lee C-H, Faltynek CR, Jarvis MF: **Effects of A-317491, a novel and selective P2X3/P2X2/3 receptor antagonist, on neuropathic, inflammatory and chemogenic nociception following intrathecal and intraplantar administration.** *Br J Pharmacol* 2003, **140**:1381–8.

28. Jang JH, Kim D-W, Sang Nam T, Se Paik K, Leem JW: **Peripheral glutamate receptors contribute to mechanical hyperalgesia in a neuropathic pain model of the rat.** *Neuroscience* 2004, **128**:169–76.

29. North RA: **P2X3 receptors and peripheral pain mechanisms.** *J Physiol* 2004, **554**:301–8.

30. Christoph T, Reissmüller E, Schiene K, Englberger W, Chizh B a: **Antiallodynic effects of NMDA glycine(B) antagonists in neuropathic pain: possible peripheral mechanisms.** *Brain Res* 2005, **1048**:218–27.

31. Gangadharan V, Wang R, Ulzhöfer B, Luo C, Bardoni R, Bali KK, Agarwal N, Tegeder I, Hildebrandt U, Nagy GG, Todd AJ, Ghirri A, Häussler A, Sprengel R, Seeburg PH, Macdermott AB, Lewin GR, Kuner R: **Peripheral calcium-permeable AMPA receptors regulate chronic inflammatory pain in mice**. *J Clin Invest* 2011, **121**:1608–1623.

32. Kung L-H, Gong K, Adedoyin M, Ng J, Bhargava A, Ohara PT, Jasmin L: **Evidence for glutamate as a neuroglial transmitter within sensory ganglia.** *PLoS One* 2013, **8**:e68312.

33. Gong K, Kung L-H, Magni G, Bhargava A, Jasmin L: **Increased response to glutamate in small diameter dorsal root ganglion neurons after sciatic nerve injury.** *PLoS One* 2014, **9**:e95491.

34. Nobbio L, Sturla L, Fiorese F, Usai C, Basile G, Moreschi I, Benvenuto F, Zocchi E, De Flora A, Schenone A, Bruzzone S: **P2X7-mediated increased intracellular calcium causes functional derangement in Schwann cells from rats with CMT1A neuropathy.** *J Biol Chem* 2009, **284**:23146–58.

35. Barry U, Zuo Z: **Opioids: old drugs for potential new applications.** *Curr Pharm Des* 2005, **11**:1343–50.

36. Moldavan MG, Irwin RP, Allen CN: **Presynaptic GABA(B) receptors regulate retinohypothalamic tract synaptic transmission by inhibiting voltage-gated Ca2+ channels.** *J Neurophysiol* 2006, **95**:3727–41.

37. Sehgal N, Smith HS, Manchikanti L: **Peripherally acting opioids and clinical implications for pain control.** *Pain Physician* 2011, **14**:249–58.

38. Pan H-L, Wu Z-Z, Zhou H-Y, Chen S-R, Zhang H-M, Li D-P: **Modulation of pain transmission by G-protein-coupled receptors.** *Pharmacol Ther* 2008, **117**:141–61.

39. Si JQ, Li ZW: **Inhibition by baclofen of NMDA-activated current in rat dorsal root ganglion neurons.** *Zhongguo Yao Li Xue Bao* 1999, **20**:324–8.

40. Takeda M, Ikeda M, Takahashi M, Kanazawa T, Nasu M, Matsumoto S: **Suppression of ATP-induced excitability in rat small-diameter trigeminal ganglion neurons by activation of GABAB receptor.** *Brain Res Bull* 2013, **98**:155–62.

41. Blednov Y a, Stoffel M, Alva H, Harris R a: **A pervasive mechanism for analgesia: activation of GIRK2 channels.** *Proc Natl Acad Sci U S A* 2003, **100**:277–82.

42. Tikoo R, Zanazzi G, Shiffman D, Salzer J, Chao M V: **Cell cycle control of Schwann cell proliferation: role of cyclin-dependent kinase-2.** *J Neurosci* 2000, **20**:4627–34.

43. Cai Q, Qiu C-Y, Qiu F, Liu T-T, Qu Z-W, Liu Y-M, Hu W-P: **Morphine inhibits acid-sensing ion channel currents in rat dorsal root ganglion neurons.** *Brain Res* 2014, **1554**:12–20.

44. Shaqura M, Khalefa BI, Shakibaei M, Zöllner C, Al-Khrasani M, Fürst S, Schäfer M, Mousa SA: **New insights into mechanisms of opioid inhibitory effects on capsaicin-**

induced TRPV1 activity during painful diabetic neuropathy. *Neuropharmacology* 2014, **85C**:142–150.

45. Chao D, Balboni G, Lazarus LH, Salvadori S, Xia Y: **Na+ mechanism of delta-opioid receptor induced protection from anoxic K+ leakage in the cortex.** *Cell Mol Life Sci* 2009, **66**:1105–15.

46. Jeong NY, Shin YH, Jung J: **Neuropathic pain in hereditary peripheral neuropathy.** *J Exerc Rehabil* 2013, **9**:397–9.

47. Laurà M, Hutton EJ, Blake J, Lunn MP, Fox Z, Pareyson D, Solari A, Radice D, Koltzenburg M, Reilly MM: **Pain and small fiber function in charcot-marie-tooth disease type 1A.** *Muscle Nerve* 2014:1–6.

48. Dave KR, Lange-Asschenfeldt C, Raval AP, Prado R, Busto R, Saul I, Pérez-Pinzón M a: **Ischemic preconditioning ameliorates excitotoxicity by shifting glutamate/gamma-aminobutyric acid release and biosynthesis.** *J Neurosci Res* 2005, **82**:665–73.

49. Zhou W, Zhu X, Zhu L, Cui YY, Wang H, Qi H, Ren QS, Chen HZ: **Neuroprotection of muscarinic receptor agonist pilocarpine against glutamate-induced apoptosis in retinal neurons.** *Cell Mol Neurobiol* 2008, **28**:263–75.

50. Zhu M, Li M, Tian X, Ou X, Zhu C, Guo J: **Neuroprotective role of delta-opioid receptors against mitochondrial respiratory chain injury.** *Brain Res* 2009, **1252**:183–91.

51. Chao D, Xia Y: **Ionic storm in hypoxic/ischemic stress: can opioid receptors subside it?** *Prog Neurobiol* 2010, **90**:439–70.

52. Iglesias M: **μ-opioid receptor activation prevents apoptosis following serum withdrawal in differentiated SH-SY5Y cells and cortical neurons via phosphatidylinositol 3-kinase**. *Neuropharmacology* 2003, **44**:482–492.

53. Tu H, Xu C, Zhang W, Liu Q, Rondard P, Pin J-P, Liu J: **GABAB receptor activation protects neurons from apoptosis via IGF-1 receptor transactivation.** *J Neurosci* 2010, **30**:749–59.

54. Olianas MC, Dedoni S, Onali P: **Signaling pathways mediating phosphorylation and inactivation of glycogen synthase kinase-3β by the recombinant human δ-opioid receptor stably expressed in Chinese hamster ovary cells.** *Neuropharmacology* 2011, **60**:1326–36.

55. Feng Y, Chao D, He X, Yang Y, Kang X, H Lazarus L, Xia Y: **A novel insight into neuroprotection against hypoxic/ischemic stress.** *Sheng Li Xue Bao* 2009, **61**:585–92.

56. Lankford KL, Arroyo EJ, Liu C-N, Somps CJ, Zorbas M a, Shelton DL, Evans MG, Hurst SI, Kocsis JD: **Sciatic nerve regeneration is not inhibited by anti-NGF antibody treatment in the adult rat.** *Neuroscience* 2013, **241**:157–69.

57. Hayashida K-I, Bynum T, Vincler M, Eisenach JC: **Inhibitory M2 muscarinic receptors are upregulated in both axotomized and intact small diameter dorsal root ganglion cells after peripheral nerve injury.** *Neuroscience* 2006, **140**:259–68.

58. Zhang Y, Zhang L, Wang F, Zhang Y, Wang J, Qin Z, Jiang X, Tao J: **Activation of M3 muscarinic receptors inhibits T-type Ca(2+) channel currents via pertussis**

toxin-sensitive novel protein kinase C pathway in small dorsal root ganglion neurons. *Cell Signal* 2011, **23**:1057–67.

59. Giordano G, Li L, White CC, Farin FM, Wilkerson HW, Kavanagh TJ, Costa LG: **Muscarinic receptors prevent oxidative stress-mediated apoptosis induced by domoic acid in mouse cerebellar granule cells.** *J Neurochem* 2009, **109**:525–38.
