# Supplementary material for: Polytherapy with a combination of three repurposed drugs (PXT3003) down-regulates Pmp22 over-expression and improves myelination, axonal and functional parameters in models of CMT1A neuropathy
Source: Orphanet J Rare Dis. 2014 Dec 10;9:201. doi: 10.1186/s13023-014-0201-x (PMC4279797; doi:10.1186/s13023-014-0201-x)
Supplement: Additional file 2: — Motor performances are significantly affected in CMT1A rats. [file 13023_2014_201_MOESM2_ESM.pdf]

Additional file 2

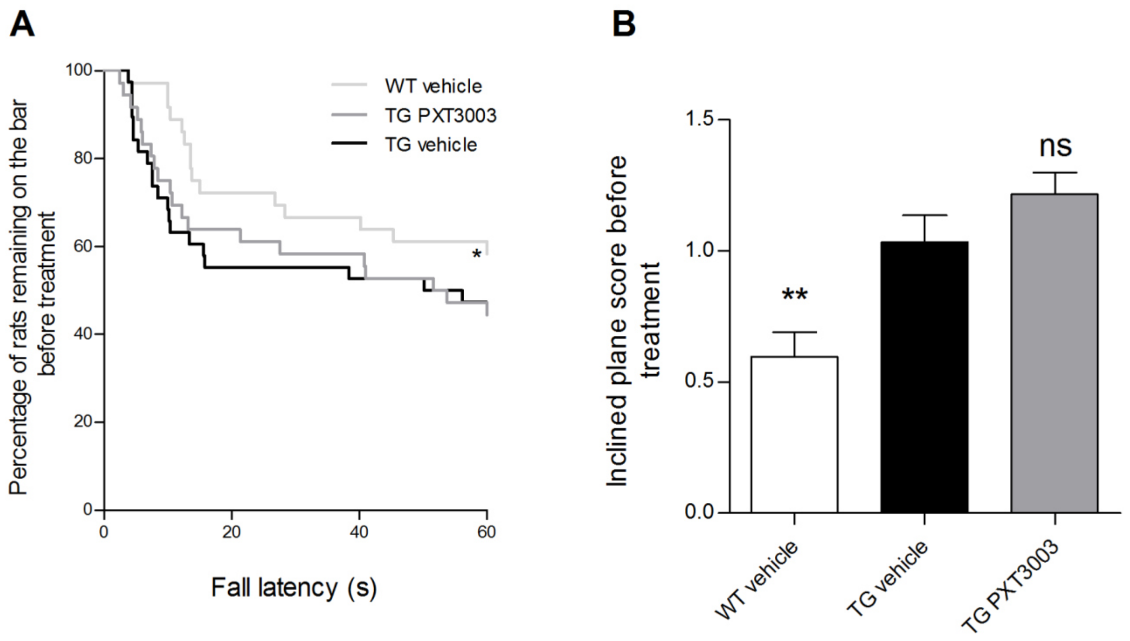

Supporting Figure 4. Motor performances are significantly affected in CMT1A rats.

(A) A 9-week oral treatment with the PXT3003 combination (BCL 30 µg/kg, NTX 3.5 µg/kg and SRB 1.05 mg/kg) demonstrated that motor performances of WT rats in the bar test were significantly better than performances of CMT TG rats. The bar test showed no difference between the TG vehicle and the TG group before the start of the treatment (*n* = 36 - 38 per group). **(B)** The inclined plane score of WT rats was significantly better compared to the score of CMT TG rats. Inclined plane scores of TG vehicle and TG rats before the treatment were not different (*n* = 36 - 38 per group). * $P < 0.05$, ** $P < 0.01$ vs TG vehicle; ANOVA with Dunnett's test. Data are shown as mean + SEM.
