# Supplementary material for: Polytherapy with a combination of three repurposed drugs (PXT3003) down-regulates Pmp22 over-expression and improves myelination, axonal and functional parameters in models of CMT1A neuropathy
Source: Orphanet J Rare Dis. 2014 Dec 10;9:201. doi: 10.1186/s13023-014-0201-x (PMC4279797; doi:10.1186/s13023-014-0201-x)
Supplement: Additional file 3: — Myelination and CMAP are unchanged after PXT3003 treatment. [file 13023_2014_201_MOESM3_ESM.pdf]

Additional file 3

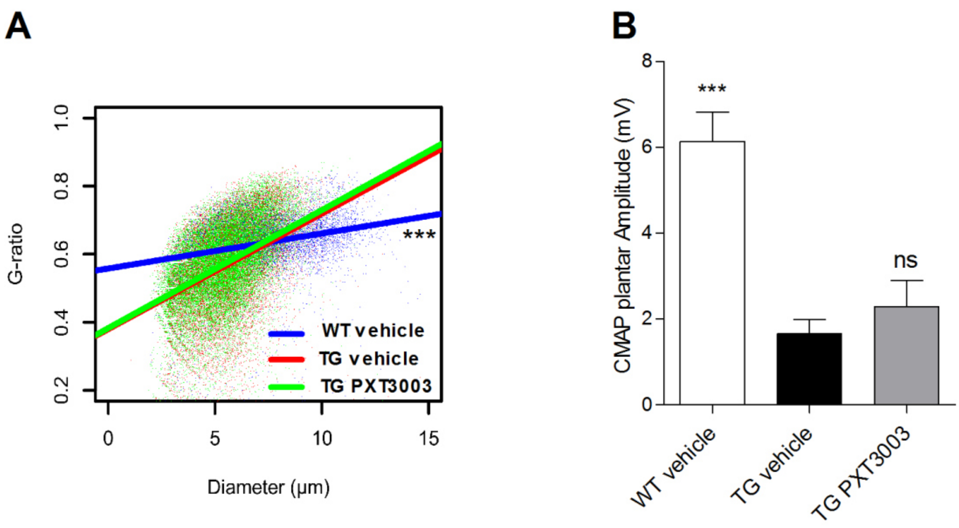

Supporting Figure 5. Myelination and amplitude of CMAP are unchanged after PXT3003 treatment.

(A) The distribution of g-ratio (ratio of the inner axonal diameter to the total outer) with respect to the axon diameter was impaired in TG vehicle. PXT3003 treatment had no effect on this distribution of g-ratio. *n* = 12, 12 and 15 for respectively WT vehicle, TG vehicle and TG PXT3003 groups. **(B)** The amplitude of plantar CMAP was significantly decreased in TG vehicle group; 8 months of PXT3003 treatment had no effect on this parameter. *n* = 11, 9 and 7 for respectively WT vehicle, TG vehicle and TG PXT3003 groups. *** $P < 0.001$ vs TG vehicle; ANOVA with Dunnett's test. Data are shown as mean + SEM.
