# Supplementary material for: Polytherapy with a combination of three repurposed drugs (PXT3003) down-regulates Pmp22 over-expression and improves myelination, axonal and functional parameters in models of CMT1A neuropathy
Source: Orphanet J Rare Dis. 2014 Dec 10;9:201. doi: 10.1186/s13023-014-0201-x (PMC4279797; doi:10.1186/s13023-014-0201-x)
Supplement: Additional file 4: — Summary of treatment characteristics in CMT1A rats. [file 13023_2014_201_MOESM4_ESM.pdf]

Additional file 4

Supporting Table 1. Summary of treatment characteristics in CMT1A rats.

OUTCOME	TREATMENT DURATION	AGE AT THE END OF TREATMENT	EFFECT	Figure
***Pmp22* GENE EXPRESSION**				
RT-Q-PCR	9 weeks	17 weeks	+	4A
**BEHAVIOUR**				
Bar Test	9 weeks	17 weeks	+	4B; 4C; Additional file 2A
Inclined Plane test	9 weeks	17 weeks	+	4D; Additional file 2B
Hot Plate	4 months	5 months	+	4E
**NERVE HISTOLOGY**				
Number of total myelinated axons	4 months	5 months	+	5A; 5D
Number of myelinated axons by diameter	4 months	5 months	+	5B; 5D
g-ratio	4 months	5 months	-	Additional file 3A
**ELECTROPHYSIOLOGY**				
CMAP	8 months	9 months	-	Additional file 3B
MNCV	8 months	9 months	+	5C

+: positive effect.

-: no effect.
