# Supplementary material for: Polytherapy with a combination of three repurposed drugs (PXT3003) down-regulates Pmp22 over-expression and improves myelination, axonal and functional parameters in models of CMT1A neuropathy
Source: Orphanet J Rare Dis. 2014 Dec 10;9:201. doi: 10.1186/s13023-014-0201-x (PMC4279797; doi:10.1186/s13023-014-0201-x)
Supplement: Additional file 5: — Nerve electrophysiology, myelin integrity and bearing function are substantially affected by the sciatic nerve crush. [file 13023_2014_201_MOESM5_ESM.pdf]

Additional file 5

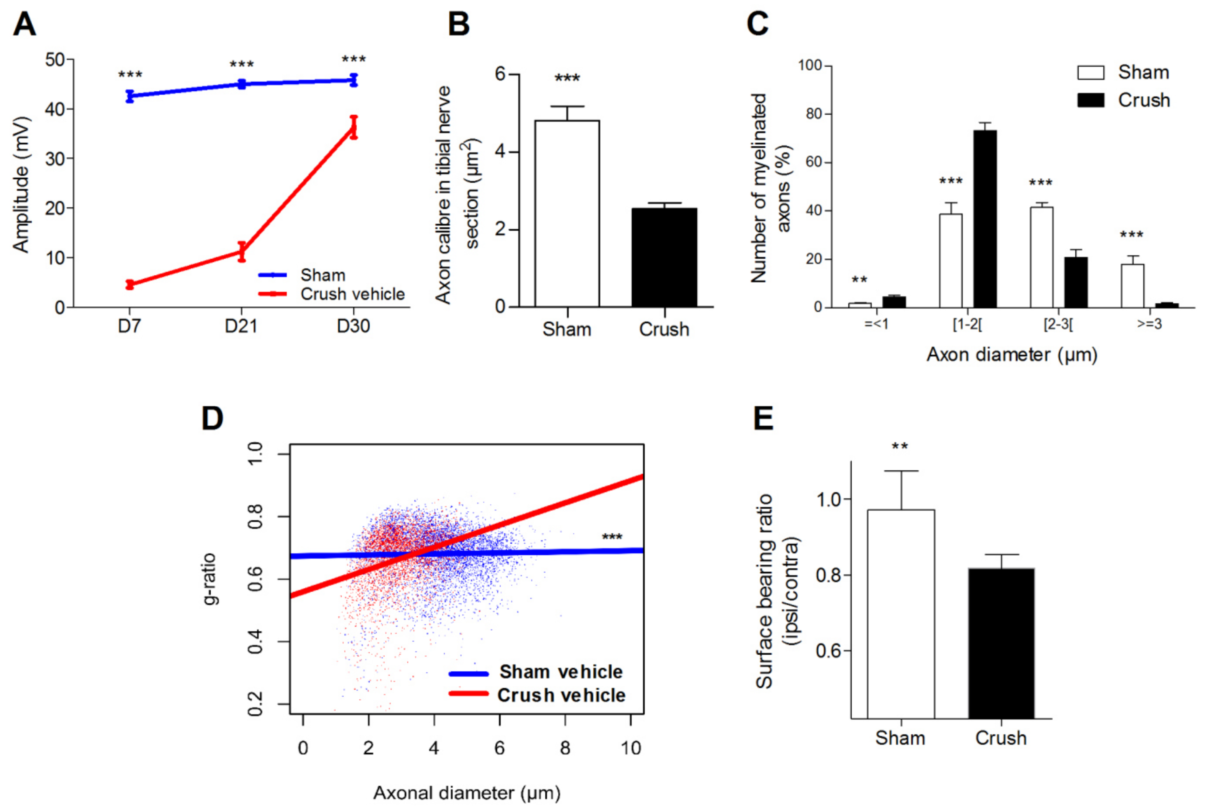

Supporting Figure 6. Nerve electrophysiology, myelin integrity and bearing function are substantially affected by the sciatic nerve crush.

(A) 7, 21 and 30 days after nerve crush, the amplitude of CMAP measured in the gastrocnemius of male mice was significantly decreased. *n* = 10 for each group. **(B)** to **(D)** 42 days after the crush, the axon calibre size measured in the tibial nerve was severely impaired **(B)**, the distribution of the number of myelinated axons in tibial nerve cross sections according to the axonal diameter was impaired **(C)** and the distribution of myelin g-ratio according to the axonal diameter in tibial nerve was modified **(D)**. *n* = 6 for each group. **(E)** Crushed mice exhibited a substantial decrease of the surface bearing ratio compared to normal mice. *n* = 10 for each group. *** $P < 0.001$ vs Crush Vehicle; *t*-test. Data are shown as mean ± SEM.
